# A Joint Model Considering Measurement Errors for Optimally Identifying Tumor Mutation Burden Threshold

**DOI:** 10.3389/fgene.2022.915839

**Published:** 2022-08-04

**Authors:** Yixuan Wang, Xin Lai, Jiayin Wang, Ying Xu, Xuanping Zhang, Xiaoyan Zhu, Yuqian Liu, Yang Shao, Li Zhang, Wenfeng Fang

**Affiliations:** ^1^ School of Computer Science and Technology, Faculty of Electronics and Information Engineering, Xi’an Jiaotong University, Xi’an, China; ^2^ School of Management, Hefei University of Technology, Hefei, China; ^3^ The Ministry of Education Key Laboratory of Process Optimization and Intelligent Decision-Making, Hefei University of Technology, Hefei, China; ^4^ Nanjing Geneseeq Technology Inc., Nanjing, China; ^5^ School of Public Health, Nanjing Medical University, Nanjing, China; ^6^ State Key Laboratory of Oncology in South China, Collaborative Innovation Center for Cancer Medicine, Sun Yat-Sen University Cancer Center, Guangzhou, China

**Keywords:** clinical immunology, stratification biomarker, tumor mutation burden, joint modeling, multiple endpoints, measurement error

## Abstract

Tumor mutation burden (TMB) is a recognized stratification biomarker for immunotherapy. Nevertheless, the general TMB-high threshold is unstandardized due to severe clinical controversies, with the underlying cause being inconsistency between multiple assessment criteria and imprecision of the TMB value. The existing methods for determining TMB thresholds all consider only a single dimension of clinical benefit and ignore the interference of the TMB error. Our research aims to determine the TMB threshold optimally based on multifaceted clinical efficacies accounting for measurement errors. We report a multi-endpoint joint model as a generalized method for inferring the TMB thresholds, facilitating consistent statistical inference using an iterative numerical estimation procedure considering mis-specified covariates. The model optimizes the division by combining objective response rate and time-to-event outcomes, which may be interrelated due to some shared traits. We augment previous works by enabling subject-specific random effects to govern the communication among distinct endpoints. Our simulations show that the proposed model has advantages over the standard model in terms of precision and stability in parameter estimation and threshold determination. To validate the feasibility of the proposed thresholds, we pool a cohort of 73 patients with non-small-cell lung cancer and 64 patients with nasopharyngeal carcinoma who underwent anti-PD-(L)1 treatment, as well as validation cohorts of 943 patients. Analyses revealed that our approach could grant clinicians a holistic efficacy assessment, culminating in a robust determination of the TMB screening threshold for superior patients. Our methodology has the potential to yield innovative insights into therapeutic selection and support precision immuno-oncology.

## Introduction

Immune checkpoint inhibitor (ICI) therapy has emerged as a promising strategy with confirmed efficacy for advanced or metastatic tumors (Bracarda et al., 2015; Motzer et al., 2015; Chiang et al., 2020; Kuryk et al., 2020; Wołącewicz et al., 2020; Majc et al., 2021). Tumor mutation burden (TMB, defined as the number of somatic mutations per mega-base) is a recognized biomarker of sensitivity to ICIs (Hellmann et al., 2018a; Cristescu et al., 2018; Bai et al., 2020; Wang et al., 2020), according to the underlying connection between the increasing number of somatic mutations and the neo-antigen that the activated T cells can recognize (Van Rooij et al., 2013), enhancing the tumor immunogenicity (Pardoll, 2012; Conway et al., 2018). A high TMB tends to trigger a favorable prognosis (Yarchoan et al., 2017; Legrand et al., 2018), which has been observed in urothelial carcinoma (Rosenberg et al., 2016), small-cell-lung cancer (Hellmann et al., 2018b), non-small-cell lung cancer (NSCLC) (Lim et al., 2015; Rizvi et al., 2015; Carbone et al., 2017; Hellmann et al., 2018c; Singal et al., 2019), and melanoma (Johnson et al., 2016; Goodman et al., 2017). TMB is a suggested test for patients undergoing immunotherapy by both NCCN and FDA (Lemery et al., 2017; Boyiadzis et al., 2018; Subbiah et al., 2020).

In clinical practices, the TMB threshold is a baseline for identifying patients with potential ICI benefits (Samstein et al., 2019). TMB thresholds are typically determined in two ways: either grouped by quartiles, which is obviously imprecise (Campesato et al., 2015; Colli et al., 2016; Riaz et al., 2017; Miao et al., 2018; Wood et al., 2020), or numerical thresholds generated from statistical tests of significance based on efficacy endpoints (Goodman et al., 2017). Notably, among these previous statistical studies, retrospective evaluations of efficacy are limited to a single dimension, most regularly the response. The primary endpoints for immuno-oncology include objective tumor response and time-to-event (TTE), where the TMB biomarker has been observed to be associated with both (Cao et al., 2019). Such diverse efficacy evaluation metrics have sparked controversy in the threshold standardization (Goodman et al., 2017). When assessments base on different endpoints over the same cohort, inconsistent thresholds arise, and clinicians are left inconclusive about which one to choose. Furthermore, clinical decisions need a comprehensive review of the diseased multifaceted efficacy rather than a single endpoint that exhibits a partial treatment effect. Therefore, there is an urgent clinical need for inference on multiple endpoints to derive a comprehensive TMB threshold. However, it is computationally challenging for two reasons. First, if several individual endpoints are to be inferred simultaneously, the intersection cannot be taken directly. Instead, some adjustment for multiple testing is required to control the familywise type I error rate (FWER) (Ristl et al., 2019). Constructing the joint distribution of different endpoints is preferable to the straightforward application of Bonferroni inequalities in terms of maximizing the utilization of available information, providing unbiased results, and allowing for statistical alpha levels closer to nominal levels while boosting the statistical power (Phillips et al., 2003; Asar et al., 2015; Guidance 2017). Secondly, the existing joint modeling studies have mostly taken a perspective on analyzing longitudinal biochemical markers within the survival analysis framework. Whereas the volatility of tumor genomic traits in immunotherapy trials is quite limited, we are more concerned with the within-subject dependence between different endpoints. Binary tumor responses conforming to the Bernoulli distribution do not satisfy the premise of a normal distribution in linear regression. The existing models have limited capacity to comprehend possible shared biologic processes on endpoints of tumor remission with survival and are not applicable to scenarios of immune efficacy investigation.

Moreover, the imprecision of TMB values is another source of threshold controversies (Wood et al., 2020). Despite the different calculation methods of TMB, the accuracy of variant callings can never reach 100% due to technical limitations (Xu et al., 2014; Alioto et al., 2015), and TMB always harbors measurement errors. Existing models neglect the difference between the actual and observed values of TMB, which lead to significant bias in statistical inference (Campesato et al., 2015; Colli et al., 2016; Goodman et al., 2017; Riaz et al., 2017; Miao et al., 2018; Wood et al., 2020). Parameter inference for statistical models is conventionally obtained by maximum likelihood estimation (MLE), and unbiasedness of the score function for likelihood (i.e., expectation equal to zero) is a critical criterion for ensuring estimate consistency. With the accurate TMB values being unascertainable, the observations TMB^∗^ ( ${\text{TMB}}^{*}=\text{TMB}+e$ ) have to be used for surrogates. The presence of its inherent random error term *e* undermines the unbiased nature of the score expectation, yielding inconsistent regression coefficient estimates. The biasing effect caused by error term confounds the proper relationship between TMB and ICI. Furthermore, naïve statistical inference assesses patient prognosis inaccurately. Thus, the final determination of TMB thresholds must be flawed, hindering accurate screening of applicable patients and closely related to the risk of adverse events to immunotherapy. Although the corrected-score methodology is associated with a measurement error (Nakamura, 1990; Novick & Stefanski, 2002; Augustin, 2004), a new algorithm should be re-inferred due to the complexity of the specific joint model. The challenge lies in the fact that the complete joint probability is essentially a complex integration without an exact analytical solution. Patients’ responses couple with the survival process, based on the random effects governing both, so that the joint score function is usually impossible to strip. It is incapable of eliminating mistakes from this joint likelihood directly. A new iterative numerical estimation procedure is required by considering the biasing impacts induced by the mis-specified TMB covariate.

Therefore, we report a generalized method for optimizing the identification of TMB-positive thresholds. Our method integrates binary response and continuous TTE endpoints to provide a comprehensive efficacy assessment, while, to our best knowledge, it is among the first statistical approaches accounting for TMB measurement errors. To verify the viability of the multi-endpoint joint model, we conducted a series of simulation experiments, and the results confirmed our superiority in the accuracy of parameter estimation and fault tolerance of threshold delineation compared with the standard separate regression model. Meanwhile, we gathered a cohort of 73 non-small-cell lung cancer (NSCLC) patients and 64 nasopharyngeal carcinoma (NPC) patients and validation cohorts of 943 patients who underwent ICI treatment to illustrate the applicability of the model across carcinomas. It is known that different cancer types and TMB calculations often yield different thresholds, but we provide here a generalized statistical method applicable for any known scenarios. The data results show that the proposed model can obtain a more comprehensive and robust TMB threshold to support therapeutic refinement for cancer patients. The source code reproduces the figures, and results can be downloaded from https://github.com/YixuanWang1120/TMB_JM.

## Materials and Methods

To comprehensively determine TMB-positivity thresholds from multifaceted efficacy analyses while considering inevitable measurement errors, we present a general approach for the simultaneous joint modeling of multiple endpoints, yielding approximately consistent statistical inference for mis-specified covariates by developing an iterative numerical estimation procedure using the corrected-score method. The observed sample information contains the patient’s clinically recorded objective response rate (ORR) and TTE endpoints, other clinical indicators (correctly specified), and the corresponding TMB observations with measurement errors. The data consist of *n* independent observations of R, T, Δ, Z, and TMB^∗^, denoting the binary tumor response outcome, continuous survival time, event indicator, vector of accurately measured covariates, and mismeasured TMB, respectively. To simplify, the additive measurement error model relates the true unobserved TMB index to the observed TMB^∗^: TMB^∗^ = TMB + *e*, where $e∼N\left(0,\sum_{e}\right)$ .

### A Joint Model Considers Binary and Continuous Endpoints

For patient i(i=1,2,…,n) , *R*_*i*_ denotes the tumor response (*R*_*i*_ = 1,0 for complete response (CR) and partial response (PR), stable disease (SD) and progressive disease (PD))and *Z*_*i*_ denotes a vector of covariates, e.g., age, gender, treatment indicator, cancer stage. Binary response outcomes are typically modeled by logistic regression whose standard form is quite well established for the immunological effectiveness analysis. *R*_*i*_ depends on *Z*_*i*_ and *TMB*_*i*_, then the mixed-effect logistical regression sub-model for the ORR endpoint is formulated as:

$$\mathrm{logit}\left(R_{i}|Z_{i},TMB_{i},b_{i};\theta \right)=\alpha_{z}^{T}Z_{i}+\alpha_{m}TMB_{i}+b_{i}$$

where *α*_*z*_ and *α*_*m*_ denote the corresponding response regression coefficients, *θ* represents all unknown parameters in the joint model, and *b*_*i*_ denotes the random effect. The exponent of the estimated parameter exp(α) for the logit regression of binary outcomes can be interpreted intuitively as the multiples of change in the odds ratio caused by a one-unit increase in the corresponding variable.

**FIGURE 1:**
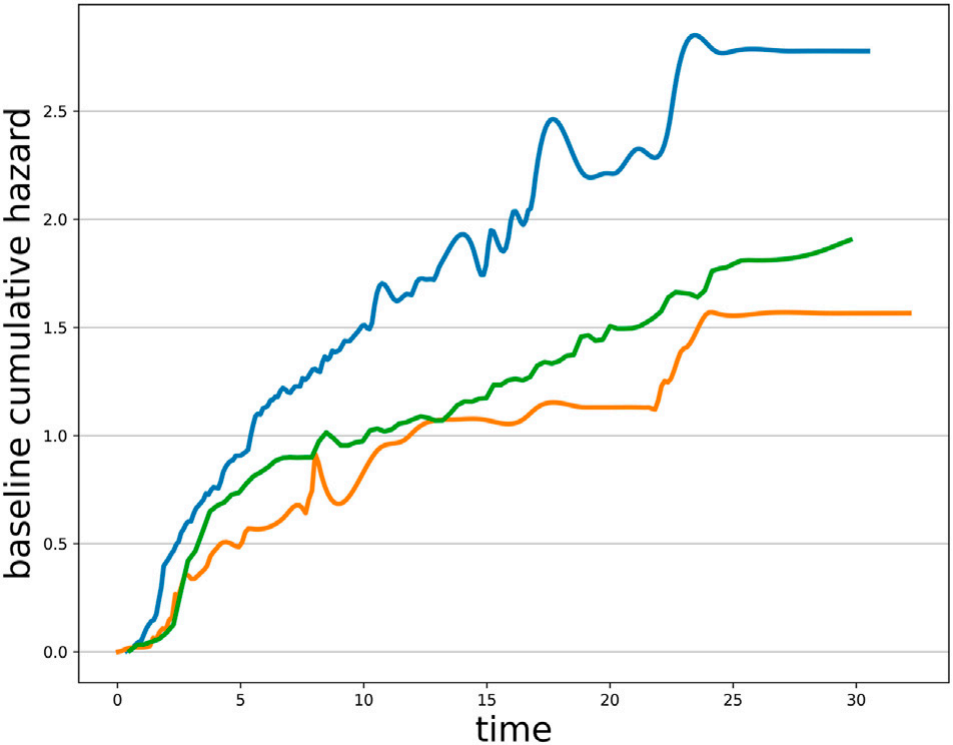
The distribution of baseline cumulative hazard for patients receiving immunotherapy.

Let *T*_*i*_ denote the observed event time (such as tumor relapses, progression, death, etc.), which is taken as the minimum of the true event time *U*_*i*_ and the censoring time *C*_*i*_, that is, $T_{i}=\min\left(U_{i},C_{i}\right)$ . Define the event indicator as $\Delta_{i}=I\left(U_{i}\leq C_{i}\right)$ , where I(⋅) is the indicator function. Here, we adopt the widely accepted Cox PH model because it focuses more on the identifying patients’ survival risk classes compared with alternative accelerated failure (AFT) models, is appropriate to the scenario of screening immunotherapy-beneficial patients in this article, and allows for more flexible baseline risk. *T*_*i*_ also depends on *Z*_*i*_, *TMB*_*i*_, unknown parameters *θ*, and random effect *b*_*i*_; then, the mixed-effect Cox PH regression sub-model for the TTE endpoint is formulated as:

$$h_{i}\left(t|Z_{i},TMB_{i},b_{i};\theta \right)=h_{0}\left(t\right)\exp\left(\beta_{z}^{T}Z_{i}+\beta_{m}TMB_{i}+b_{i}\right)$$

$$\begin{array}{l} S_{i}\left(t|Z_{i},TMB_{i},b_{i};\theta \right)=\exp\left\{-\int_{0}^{t}h_{0}\left(s\right)\exp\left(\beta_{z}^{T}Z_{i}+\beta_{m}TMB_{i}+b_{i}\right)ds\right\}\\ \;\;\;\;\;\;\;\;=\exp\left\{-{ℋ}_{0}\left(t\right)\exp\left(\beta_{z}^{T}Z_{i}+\beta_{m}TMB_{i}+b_{i}\right)\right\} \end{array}$$

where *h*(*t*) describes the instantaneous risk for an event in the time interval [*t*, *t* + *dt*) provided survival up to *t*, while *S*(*t*) represents the survival probability. *h*_0_(*t*) is referred to as *baseline hazard* and follows the Weibull distribution $h_{0}\left(t\right)=\lambda t^{\left(\lambda -1\right)}$ because the trend in the baseline cumulative hazard distribution for progression-free survival in the cohort receiving immunotherapy is consistent with the Weibull distribution with a scale parameter equal to 1 (see in Figure 1). *β*_*z*_ is the corresponding vector of covariate effect and *β*_*m*_ quantifies the TMB effect.

The maximum likelihood estimates are derived as the modes of the log-likelihood function corresponding to the joint distribution of the observed samples $D_{n}=\left\{R_{i},T_{i},\Delta_{i},Z_{i},TMB_{i},i=1,\ldots n\right\}$ . The vector $b={\left(b_{1},b_{1},\ldots ,b_{n}\right)}^{\prime }$ is the shared random effect on the respective endpoints, accounting for the intra-subject correlation between event times and individual tumor response and is assumed to follow a normal distribution $N\left(0,\sigma_{b}^{2}\right)$ . Since the random effect *b*_*i*_ accounts for the intra-subject association underlying both response and survival process, thus the two are conditionally independent given the random effect. Formally, for patient *i*, we have that:

$$p\left(R_{i},T_{i},\Delta_{i},b_{i};\theta \right)=p\left(R_{i}|b_{i};\theta \right)\cdot p\left(T_{i},\Delta_{i}|b_{i};\theta \right)\cdot p\left(b_{i};\theta \right)$$ (1)

where the likelihood of the response is:

$$\begin{array}{c} p\left(R_{i}|b_{i};\theta \right)=F{\left(\alpha_{z}^{T}Z_{i}+\alpha_{m}TMB_{i}+b_{i}\right)}^{R_{i}}{\left\{1-F\left(\alpha_{z}^{T}Z_{i}+\alpha_{m}TMB_{i}+b_{i}\right)\right\}}^{\left(1-R_{i}\right)}\\ F\left(\upsilon \right)={\left(1+e^{-\upsilon }\right)}^{-1} \end{array}$$

while the likelihood of the survival is:

$$p\left(T_{i},\Delta_{i}|b_{i};\theta \right)=h_{i}{\left(T_{i}|b_{i};\theta \right)}^{\Delta_{i}}\cdot S_{i}\left(T_{i}|b_{i};\theta \right).$$

By incorporating random effects (Barbieri et al., 2020), it is feasible to jointly model the multiple endpoints and regulate intricate correlations between response probabilities and event times. Then, the joint logarithmic likelihood can be formulated as:

$$\begin{array}{l} \ell \left(\theta \right)=\sum_{i}\log p\left(R_{i},T_{i},\Delta_{i};\theta \right)\\ \;\;=\sum_{i}\log\int p\left(R_{i}|b_{i};\theta \right)p\left(T_{i},\Delta_{i}|b_{i};\theta \right)p\left(b_{i};\theta \right)db_{i} \end{array}$$ (2)

Inference about parameters *θ* is typically based on the maximization of Eq. 2, while integrals about random effects apparently have no analytical solution. Here, we approximate ℓ(θ) based on the Laplace method, which has the advantage over other numerical integration techniques, including Gaussian Hermite quadrature and Monte Carlo (Lin et al., 2008; Rizopoulos et al., 2014), since the multiplicative form of the series can be easily unfolded by adopting the logarithmic trick, facilitating our correction of the measurement errors of the covariates later. The Laplace approximation is as follows:

$$\int_{a}^{b}e^{f\left(x\right)}dx\approx \sqrt{\frac{2\pi }{\left|f^{\prime \prime }\left(x_{0}\right)\right|}}e^{f\left(x_{0}\right)}$$

where the function *f*(*x*) has a unique global maximum at *x*_0_. So, the first-order Laplace approximation to the observed-data joint log-likelihood is:

$${\tilde{\ell }}_{i}\left(\theta ,{\hat{b}}_{i}\right)=\frac{1}{2}\log2\pi +\log p\left(R_{i}|{\hat{b}}_{i};\theta \right)+\log p\left(T_{i},\Delta_{i}|{\hat{b}}_{i};\theta \right)+\log p\left({\hat{b}}_{i};\theta \right)-\frac{1}{2}\log\left|k^{\prime \prime }\left({\hat{b}}_{i};\theta \right)\right|$$ (3)

where

$$\begin{array}{l} k\left(b_{i};\theta \right)=\log p\left(R_{i}|b_{i};\theta \right)+\log p\left(T_{i},\Delta_{i}|b_{i};\theta \right)+\log p\left(b_{i};\theta \right)\\ \;\;\;=R_{i}\left(\alpha_{z}^{T}Z_{i}+\alpha_{m}TMB_{i}+b_{i}\right)-\log\left\{1+\exp\left(\alpha_{z}^{T}Z_{i}+\alpha_{m}TMB_{i}+b_{i}\right)\right\}\\ \;\;\;+\Delta_{i}\left\{\log h_{0}\left(T_{i}\right)+\beta_{z}^{T}Z_{i}+\beta_{m}TMB_{i}+b_{i}\right\}\\ -{ℋ}_{0}\left(T_{i}\right)\exp\left(\beta_{z}^{T}Z_{i}+\beta_{m}TMB_{i}+b_{i}\right)\\ \;\;\;+\log\left(2\pi \right)/2+\log\left(\sigma_{b}\right)-b_{1}^{2}/2\sigma_{b}^{2} \end{array}$$ (4)

and the mode ${\hat{b}}_{i}$ is obtained for each patient by solving $k^{\prime }\left(b_{i}\right)=0$ with a fixed *θ*,

$$\begin{array}{l} k^{\prime }\left(b_{i};\theta \right)=\frac{\partial \log p\left(R_{i}|b_{i};\theta \right)}{\partial b_{i}}+\frac{\partial \log p\left(T_{i},\Delta_{i}|b_{i};\theta \right)}{\partial b_{i}}+\frac{\partial \log p\left(b_{i};\theta \right)}{\partial b_{i}}\\ \;\;\;\;=R_{i}-F\left(\alpha_{z}^{T}Z_{i}+\alpha_{m}TMB_{i}+b_{i}\right)+\Delta_{i}-{ℋ}_{0}\left(T_{i}\right)\exp\left(\beta_{z}^{T}Z_{i}+\beta_{m}TMB_{i}+b_{i}\right)-b_{i}\sigma_{b}^{-2} \end{array}$$ (5)

$$\begin{array}{l} \left|k^{\prime \prime }\left(b_{i};\theta \right)\right|=F\left(\alpha_{z}^{T}Z_{i}+\alpha_{m}TMB_{i}+b_{i}\right)\left\{1-F\left(\alpha_{z}^{T}Z_{i}+\alpha_{m}TMB_{i}+b_{i}\right)\right\}\\ \;\;\;\;\;+{ℋ}_{0}\left(T_{i}\right)\exp\left(\beta_{z}^{T}Z_{i}+\beta_{m}TMB_{i}+b_{i}\right)-\sigma_{b}^{-2} \end{array}$$ (6)

The difference of Eq. 3 from the previous independent standard regressions lies in that the joint assessment entails examining the endpoint correlations, where $\log p\left(R_{i}|{\hat{b}}_{i};\theta \right)$ represents the likelihood of ORR, while $\log p\left(T_{i},\Delta_{i}|{\hat{b}}_{i};\theta \right)$ represents the information on survival endpoint, and $\log p\left({\hat{b}}_{i};\theta \right)-\frac{1}{2}\log\left|k^{\prime \prime }\left({\hat{b}}_{i};\theta \right)\right|$ incorporates the within-subject dependence between two endpoints. When ${\hat{b}}_{i}=0$ , i.e., there is no correlation between the two clinical endpoints, the joint model degenerates to standard separate logistic regression and Cox PH regression.

Estimates obtained by maximizing $\tilde{\ell }\left(\theta \right)=\sum_{i}{\tilde{\ell }}_{i}\left(\theta ,{\hat{b}}_{i}\right)$ are thus approximate maximum likelihood estimates (MLEs). The maximization is accomplished by solving the equation $\Psi \left(\theta \right)=\frac{\partial \tilde{\ell }\left(\theta \right)}{\partial \theta^{\text{T}}}=0$ , Ψ(θ) is *score function*. According to the negative of the inverse Hessian matrix at MLE $\hat{\theta }$ , we obtain the standard errors for the parameter estimates $v\hat{a}r\left(\hat{\theta }\right)={\left\{-H\left(\hat{\theta }\right)\right\}}^{-1}$ , with $H\left(\hat{\theta }\right)={\left\{{-\frac{\partial \Psi \left(\theta \right)}{\partial \theta^{\text{T}}}|}_{\hat{\theta }}\right\}}^{-1}$ , and the asymptotic confidence interval is $\hat{\theta }\pm 1.96\hat{se}\left(\hat{\theta }\right)$ . It is typically easier to employ a numerical derivative routine for the calculation of Hessian matrix, such as the forward or the central difference approximation.

Based on $\hat{\theta }$ , we obtain approximately consistent and unbiased estimates of the fixed effects for TMB and the random effects symbolizing intra-subject correlations between both endpoints. With the mutually moderating random effects, the joint likelihood that a patient has a favorable prognosis can be determined. This joint probability characterizes the positive prognosis of patients with both remission of tumor lesions and prolonged survival time, which can be utilized to analyze the patient’s treatment outcome more completely. The joint probability for patient *i* is:

p(Ri=1,Ti>T0;θ^)=2π|{∂2⁡log(p(Ri=1|bi;θ^)p(Ti>T0|bi;θ^)p(bi;θ^))∂bi2}b^i|p(Ri=1|b^i;θ^)p(Ti>T0|b^i;θ^)p(b^i;θ^) (7)

where *T*_0_ is a pre-specified survival time.

Based on the joint probabilities that characterized the positive prognosis of the patients, we rank them and then label the populations to be analyzed according to the proportion of patients who would potentially benefit for ICI. Ultimately, the proposed joint model can stratify patients into two subgroups according to their TMB levels and the positive prognosis labels using the receiver operating characteristic curve (ROC) to balance the classification performance. Thresholds for the low- and high-TMB groups are selected from the local optima across a range of clinically meaningful values by Yoden Index.

The complete TMB threshold identification procedure based on the aforementioned joint model solved by Laplace approximation is given in Algorithm 1.

Algorithm 1 Identifying TMB threshold without measurement errors

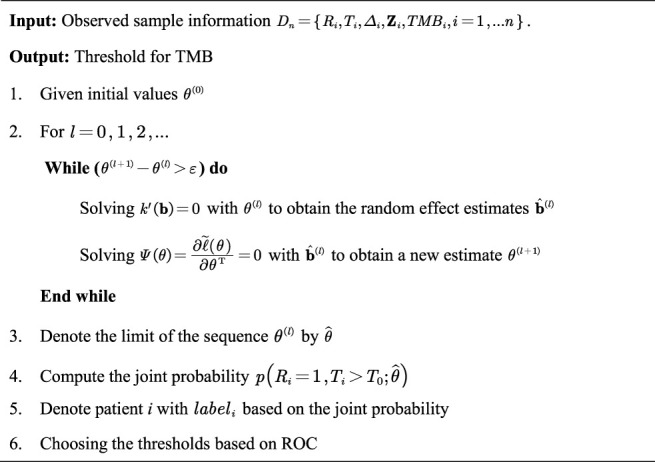

### Bias Arising From Measurement Error

Here, we further investigate the negative impact of measurement errors in TMB. The score function in Section 2.1 is unbiased. Base on Eqs. 3, 6, $\theta^{T}=\left[\theta_{R}^{T},\theta_{T}^{T},\theta_{b}\right]$ , $\theta_{R}={\left[\alpha_{z}^{T},\alpha_{m}\right]}^{T}$ , $\theta_{T}={\left[\lambda ,\beta_{z}^{T},\beta_{m}\right]}^{T}$ , $\theta_{b}=\sigma_{b}$ , we have:

$$\begin{array}{l} \Psi_{i}\left(R_{i},T_{i},\Delta_{i},Z_{i},TMB_{i};\Theta \right)=\frac{\partial {\tilde{\ell }}_{i}\left(\theta ,{\hat{b}}_{i}\right)}{\partial \theta^{T}}=\Psi_{R,i}\left(\theta \right)+\Psi_{T,i}\left(\theta \right)+\Psi_{b,i}\left(\theta \right)\\ \Psi_{i}\left(\theta_{R}\right)=\Psi_{R,i}\left(\theta_{R}\right)+\Psi_{b,i}\left(\theta_{R}\right)\\ =\left\{R_{i}-F\left(\alpha_{z}^{T}Z_{i}+\alpha_{m}TMB_{i}+{\hat{b}}_{i}\right)-\frac{1}{2}\frac{\exp\left(\alpha_{z}^{T}Z_{i}+\alpha_{m}TMB_{i}+{\hat{b}}_{i}\right)\left\{1-\exp\left(\alpha_{z}^{T}Z_{i}+\alpha_{m}TMB_{i}+{\hat{b}}_{i}\right)\right\}}{\left|k^{\prime \prime }\left({\hat{b}}_{i};\theta \right)\right|{\left\{1+\exp\left(\alpha_{z}^{T}Z_{i}+\alpha_{m}TMB_{i}+{\hat{b}}_{i}\right)\right\}}^{3}}\right\}\left(\begin{array}{c} Z_{i}\\ TMB_{i} \end{array}\right)\\ \Psi_{i}\left(\lambda \right)=\Psi_{T,i}\left(\lambda \right)+\Psi_{b,i}\left(\lambda \right)\\ \;\;\;=\Delta_{i}\left(\lambda^{-1}+\log T_{i}\right)-T_{i}^{\lambda }\log T_{i}\exp\left(\beta_{z}^{T}Z_{i}+\beta_{m}TMB_{i}+{\hat{b}}_{i}\right)+\frac{1}{2}\frac{T_{i}^{\lambda }\log T_{i}\exp\left(\beta_{z}^{T}Z_{i}+\beta_{m}TMB_{i}+{\hat{b}}_{i}\right)}{\left|k^{\prime \prime }\left({\hat{b}}_{i};\theta \right)\right|}\\ \Psi_{i}\left(\beta_{z}^{T},\beta_{m}\right)=\Psi_{T,i}\left(\beta_{z}^{T},\beta_{m}\right)+\Psi_{b,i}\left(\beta_{z}^{T},\beta_{m}\right)\\ =\left\{\Delta_{i}-T_{i}^{\lambda }\exp\left(\beta_{z}^{T}Z_{i}+\beta_{m}TMB_{i}+{\hat{b}}_{i}\right)+\frac{1}{2}\frac{T_{i}^{\lambda }\exp\left(\beta_{z}^{T}Z_{i}+\beta_{m}TMB_{i}+{\hat{b}}_{i}\right)}{\left|k^{\prime \prime }\left({\hat{b}}_{i};\theta \right)\right|}\right\}\left(\begin{array}{c} Z_{i}\\ TMB_{i} \end{array}\right)\\ \Psi_{i}\left(\sigma_{b}\right)=\Psi_{b,i}\left(\sigma_{b}\right)=-\sigma_{b}^{-1}+{\hat{b}}_{1}^{2}\cdot \sigma_{b}^{-3}+{\left(\sigma_{b}\left|k^{\prime \prime }\left({\hat{b}}_{i};\theta \right)\right|\right)}^{-1} \end{array}$$ (8)

where $\Psi_{R_{i}}\left(\theta \right)$ represents the score of ORR, $\Psi_{T_{i}}\left(\theta \right)$ represents the score on the survival endpoint, and $\Psi_{b_{i}}\left(\theta \right)$ represents the score of random effect.

The parameter $\hat{\theta }$ relating R, T, Δ, Z, and TMB is approximately consistent by satisfying $\sum_{i=1}^{n}\Psi \left(R_{i},T_{i},\Delta_{i},Z_{i},TMB_{i};\hat{\theta }\right)=0$ , where the score function Ψ is conditionally unbiased for the approximate likelihood:

$$E\left\{\Psi \left(R_{i},T_{i},\Delta_{i},Z_{i},TMB_{i};\text{Θ}\right)\right\}=0\;i=1,\ldots ,n.$$ (9)

What will happen when measurement error exists? We assume the observed TMB^∗^ is subject to the measurement error model: $TMB_{i}^{*}=TMB_{i}+e_{i},i=1,\ldots ,n$ . The error term *e*_*i*_ is independent and identically normal distributed with mean zero and known variance $\sigma_{e}^{2}$ , and is independent of *R*_*i*_, *T*_*i*_, Δ_*i*_, and **Z**_*i*_. Because true *TMB* is not observed and hence the true-data score function cannot be used for parameter estimation from the perspective of inconsistency $E\left\{\Psi \left({\text{TMB}}^{*};\Theta \right)\right\}=E\left\{\Psi \left(\text{TMB}+e;\Theta \right)\right\}\neq 0$ .

As a more specific illustration, we consult the part of survival function:

$$\begin{array}{l} E\left\{\Delta_{i}Z_{i}-T_{i}^{\lambda }\exp\left(\beta_{z}^{T}Z_{i}+\beta_{m}TMB_{i}^{*}+{\hat{b}}_{i}\right)Z_{i}\right\}\\ =\Delta_{i}Z_{i}-T_{i}^{\lambda }E\left\{\exp\left(\beta_{z}^{T}Z_{i}+\beta_{m}TMB_{i}+\beta_{m}e_{i}+{\hat{b}}_{i}\right)\right\}Z_{i}\\ =\Delta_{i}Z_{i}-T_{i}^{\lambda }\exp\left(\beta_{z}^{T}Z_{i}+\beta_{m}TMB_{i}+{\hat{b}}_{i}\right)Z_{i}E\left\{\exp\left(\beta_{m}e_{i}\right)\right\}\\ \neq 0 \end{array}$$ (10)

The additional term $E\left\{\exp\left(\beta_{m}e_{i}\right)\right\}$ on the scoring function is generated by the measurement error, leading the naïve estimator to be biased apparently. As for the response score and distribution of random effects, $F\left(\alpha_{z}^{T}Z_{i}+\alpha_{m}TMB_{i}+\alpha_{m}e_{i}+{\hat{b}}_{i}\right)$ and $\frac{1}{2}\log\left|\left(k^{\prime \prime }\left({\hat{b}}_{i},TMB_{i},e_{i};\theta \right)\right)\right|$ are also subject to the negative impact of the error term with non-zero expectations:

$$E\left\{F\left(\alpha_{z}^{T}Z_{i}+\alpha_{m}TMB_{i}+\alpha_{m}e_{i}+{\hat{b}}_{i}\right)\right\}=\int_{-\infty }^{+\infty }F\left(\alpha_{z}^{T}Z_{i}+\alpha_{m}TMB_{i}+\alpha_{m}e_{i}+{\hat{b}}_{i}\right)p\left(e_{i}\right)de_{i}$$

due to the function F(⋅) is not axisymmetric about the origin. The presence of the inevitable random error term ***e*** undermines the unbiased nature of the score expectation.

### Correction of TMB Measurement Error for Threshold Optimization

To reduce the biasing effect caused by measurement errors and obtain a more robust TMB threshold, we integrated the widely applicable corrected score with the joint model, resulting in approximately consistent estimators based on the observed data. A corrected score is a function $\Psi_{c}^{*}$ of the observed data having the important property that

$$E\left\{\Psi_{c}^{*}\left(R_{i},T_{i},\Delta_{i},Z_{i},TMB_{i}^{*};\Theta \right)|R_{i},T_{i},\Delta_{i},Z_{i},TMB_{i}\right\}=\Psi \left(R_{i},T_{i},\Delta_{i},Z_{i},TMB_{i};\Theta \right)$$ (11)

which is conditionally unbiased for the true-data score function according to the property of conditional expectation, $E\left\{\Psi_{c}^{*}\left(R_{i},T_{i},\Delta_{i},Z_{i},TMB_{i}^{*};\Theta \right)\right\}=0$ . The corrected scores provide an approach to reducing bias incurred by a covariate measurement error. Thus, $\Psi_{c}^{*}$ possesses a consistent, asymptotically normally sequence of solutions for $\sum_{i=1}^{n}\Psi_{c}^{*}\left(R_{i},T_{i},\Delta_{i},Z_{i},TMB_{i}^{*};\Theta \right)=0$ (Nakamura, 1990; Carroll et al., 2006).

Based on Eqs. 4 and 8 and the property of corrected score, we derive a correct $k_{c}^{\prime }\left(b_{i}\right)$ for the random effect estimator, and a corrected score $\Psi_{c}^{*}$ for the *ideal likelihood score* Ψ . The corrected scores are defined as follows.

Let

kc′(bi)=Ri−J−1∑j−1JRe{F(αzTZi+αm,TMB˜j,i∗+bi)}+Δi−Tiλm(βm)−1⁡exp(βzTZi+βmTMBi∗+bi)−biσb2 (12)

where the complex variate ${\tilde{TMB}}_{j,i}^{*}=TMB_{i}^{*}+\sqrt{-1}\xi_{j,i}$ , and $\xi_{j,i}$ is a normal random vector with zero mean and variance $\sigma_{e}$ . Then, $k^{\prime }\left(b_{i}\right)$ is the corrected-score function for $k^{\prime }\left(b_{i}\right)$ . The proof can be found in the Supplementary Material.

Furthermore, we obtain the joint corrected-score $\Psi_{c}^{*}\left(R,T,\Delta ,Z,\mathrm{TM}B^{*};\Theta \right)={\left[\Psi_{c}^{*}\left(\theta_{R}^{T}\right),\Psi_{c}^{*}\left(\theta_{T}^{T}\right),\Psi_{c}^{*}\left(\theta_{b}\right)\right]}^{T}$ , where $\Psi_{c}^{*}\left(\theta_{R}\right)=\Psi_{R\_c}^{*}\left(\theta_{R}\right)+\Psi_{b\_c}^{*}\left(\theta_{R}\right)$ , $\Psi_{c}^{*}\left(\theta_{T}\right)=\Psi_{T\_c}^{*}\left(\theta_{T}\right)+\Psi_{b\_c}^{*}\left(\theta_{T}\right)$ , $\Psi_{c}^{*}\left(\theta_{b}\right)=\Psi_{b\_c}^{*}\left(\theta_{b}\right)$ ,

ΨR_c,i∗(θR)=Ri(ZiTMBi∗)−J−1∑j=1JRe{F(αzTZi+αmTMBj,i∗˜+b^i)(ZiTMBi∗˜)}ΨT_c,i∗(λ)=Δi(λ−1+log⁡Ti)−Tiλ⁡log⁡Ti⁡exp(βzTZi+βmTMBi∗+b^i)m(βm)−1ΨT_c,i∗(βzT)=[Δi−Tiλ⁡exp(βzTZi+βmTMBi∗+b^i)m(βm)−1]ZiΨT_c,i∗(βm)=ΔiTMBi∗−Tiλ⁡exp(βzTZi+βmTMBi∗+b^i)m(βm)−1[TMBi∗−m(βm)−1{∂m(βm)∂βm}] (13)

Ψb_c,i(θR)=−12|kc″(b^i;θ)|J−1∑j=1JRe{exp(αzTZi+αmTMBj,i∗˜+b^i){1−exp(αzTZi+αmTMBj,i∗˜+b^i)}{1+exp(αzTZi+αmTMBj,i∗˜+b^i)}3}(ZiTMB˜j,i∗)Ψb_c,i(λ)=Tiλ⁡log⁡Ti⁡exp(βzTZi+βmTMBi∗+b^i)m(βm)−12|kc″(b^i;θ)|Ψb_c,i(βzT)=Tiλ⁡exp(βzTZi+βmTMBi∗+b^i)m(βm)−12|kc″(b^i;θ)|ZiΨb_c,i(βm)=Tiλ⁡exp(βzTZi+βmTMBi∗+b^i)m(βm)−12|kc″(b^i;θ)|[TMBi∗−m(βm)−1{∂m(βm)∂βm}]Ψb_c,i(σb)=−σb−1+b^i2⋅σb−3+σb−1|kc″(b^i;θ)|−1 (14)

We present the joint corrected scores based on the complex variable simulation extrapolation and the property of Eq. 11. Eq. 13 contains $\Psi_{R\_c}^{*}\left(\theta \right)$ representing the corrected score for ORR, which follows the complex variable simulation extrapolation for logistic regression (see Lemma 3 in the Supplementary Material), while $\Psi_{T\_c}^{*}\left(\theta \right)$ represents the corrected score for TTE satisfying the property of Eq. 11 (see Lemma 2 in the Supplementary Material). Then, based on the specificity of joint modeling, additional $\Psi_{b\_c}^{*}\left(\theta \right)$ needs to be considered, which represents the difference between the standard correction and the joint model correction. Then, $\Psi_{c}^{*}$ is the corrected-score function with the proof in the Supplementary Material. Consistency is achieved by virtue of the fact that the estimators are M-estimators whose score functions are unbiased in the presence of measurement error. The critical challenges of inferring the joint model are the random effects that characterize within-subject correlations. In the presence of measurement error, we need to correct the score functions of the random effects $k^{\prime }\left({\hat{b}}_{i}\right)$ to ensure the unbiasedness of their estimates before dealing with complex joint score functions without exact solutions by $k_{c}^{\prime \prime }\left({\hat{b}}_{i}\right)$ as well as Monte Carlo extrapolation, which is the gap in the existing literature addressed in this article. Solving the equations $k_{c}^{\prime }\left({\hat{b}}_{i}\right)=0$ and $\sum_{i=1}^{n}\Psi_{c}^{*}\left(R_{i},T_{i},\Delta_{i},Z_{i},TMB_{i}^{*};\Theta \right)=0$ by the Newton–Raphson iteration, it is ultimately possible to yield the approximately consistent estimators $\tilde{\theta }$ for mis-specified covariates and $\tilde{b}$ for random effects.

The complete TMB threshold identification procedure based on the aforementioned Laplace approximation and corrected score is given in Algorithm 2.

Algorithm 2 Identifying TMB threshold with measurement errors

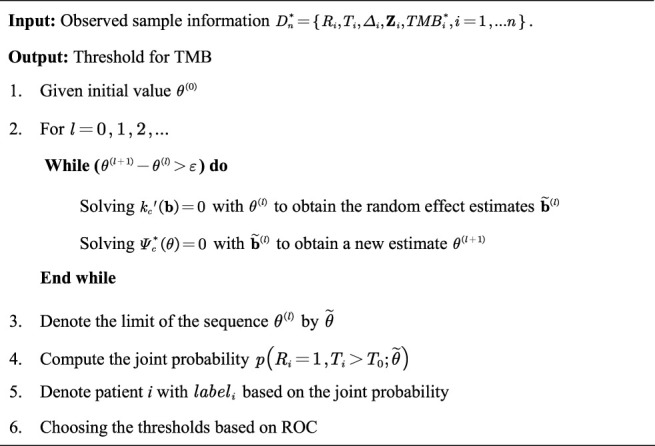

## Experiments and Results

### Simulation Study

In order to assess the performance of the proposed joint model with the corrected-score function, we conducted a series of simulation studies whose primary objective was to assess the fixed effect coefficient estimates and the variance of the random effects. Data are simulated in an oncology trial context, with random effects correlated among patients’ multiple endpoints. In the simulations, we assume 200 patients, i.e., i=1,…,200 . For each patient *i*, we generate the random effects *b*_*i*_ from a normal distribution with zero mean, variance $\sigma_{b}$ . We consider three distinct tumor response states CR&PR (*R*_*i*_ = 1), SD & PD (*R*_*i*_ = 0). The response data are generated based on the logistic probability, $\pi_{1}=F\left(\alpha_{z}^{T}Z_{i}+\alpha_{m}TMB_{i}+b_{i}\right)$ , $\pi_{0}=1-\pi_{1}$ . The event time for the patient is generated from the probability density function $h_{0}\left(t\right)\exp\left(\beta_{z}^{T}Z_{i}+\beta_{m}TMB_{i}+b_{i}\right)S_{0}ɛ{\left(t\right)}^{\exp\left(\beta_{z}^{T}Z_{i}+\beta_{m}TMB_{i}+b_{i}\right)}$ , where the baseline hazard is assumed to follow the Weibull distribution with the shape parameter equal to 1.0. Censoring time *C* is generated from the uniform distribution *U* (0, 8).

Furthermore, we set $\alpha_{z}=-1.8,\alpha_{m}=-0.3$ , $\lambda =-1.0,\beta_{z}=-2.2$ , and $\beta_{m}=-0.4$ , $\sigma_{b}=-1.0$ . The variance for the error term is set to be 0.5, 0.75, and 1.0, respectively, in order to evaluate the performance of the estimators with different measurement error levels. With the specified parameters, for each dataset, based on the joint model, the true-data estimator, the naive estimator and the correct-score estimator with Monte Carlo approximation J = 10 were computed 1,000 replications. As a comparison, we also based the standard regression models; the true-data estimator and the naive estimator were computed. Results of the simulations are presented in Table 1. We report the fitted value, average bias, SD, and SE for each parameter, where SD and SE are defined as the standard error of the estimates over 1,000 simulations and the average of the standard error of the estimates, respectively.

**TABLE 1:** Comparisons of bias and standard errors of estimators between joint model with standard model with varying measurement errors.

Model and estimator	Coef	Fitted value	Average Bias	SD	SE
Joint Model True-data estimator	*α*	−1.857	0.057	0.547	0.508
	*α*_*m*_	**0.277**	**0.023**	**0.099**	**0.093**
	λ	0.995	0.005	0.084	0.066
	*β*	2.109	0.091	0.315	0.302
	*β*_*m*_	−**0.427**	**0.027**	**0.068**	**0.060**
	*σ*_*b*_	0.981	0.019	0.167	0.064
Joint Model Correct-score estimator *σ*_*err*_ = 0.5	*α*	−1.854	0.054	0.495	0.503
	*α*_*m*_	**0.278**	**0.022**	**0.091**	**0.092**
	λ	0.989	0.011	0.083	0.066
	*β*	2.117	0.083	0.373	0.296
	*β*_*m*_	−**0.425**	**0.025**	**0.081**	**0.060**
	*σ*_*b*_	0.956	0.044	0.148	0.064
Joint Model Correct-score estimator *σ*_*err*_ = 0.75	*α*	−1.798	0.002	0.477	0.502
	*α*_*m*_	**0.271**	**0.029**	**0.090**	**0.091**
	λ	0.979	0.021	0.075	0.066
	*β*	2.108	0.092	0.370	0.293
	*β*_*m*_	−**0.418**	**0.018**	**0.076**	**0.059**
	*σ*_*b*_	0.922	0.078	0.124	0.062
Joint Model Correct-score estimator *σ*_*err*_ = 1.0	*α*	−1.790	0.010	0.444	0.498
	*α*_*m*_	**0.274**	**0.026**	**0.081**	**0.090**
	λ	0.972	0.028	0.074	0.065
	*β*	2.027	0.173	0.362	0.288
	*β*_*m*_	−**0.390**	**0.010**	**0.074**	**0.058**
	*σ*_*b*_	0.893	0.107	0.106	0.061
Joint Model Naive estimator *σ*_*err*_ = 0.5	*α*	−1.685	0.115	0.409	0.482
	*α*_*m*_	**0.257**	**0.043**	**0.069**	**0.087**
	λ	0.947	0.053	0.072	0.064
	*β*	1.757	0.443	0.326	0.281
	*β*_*m*_	−**0.344**	**0.056**	**0.060**	**0.056**
	*σ*_*b*_	0.854	0.146	0.105	0.059
Joint Model Naive estimator *σ*_*err*_ = 0.75	*α*	−1.591	0.209	0.403	0.465
	*α*_*m*_	**0.246**	**0.054**	**0.066**	**0.082**
	λ	0.924	0.076	0.064	0.063
	*β*	1.436	0.764	0.296	0.264
	*β*_*m*_	−**0.268**	**0.132**	**0.047**	**0.051**
	*σ*_*b*_	0.788	0.212	0.070	0.056
Joint Model Naive estimator *σ*_*err*_ = 1.0	*α*	−1.585	0.215	0.483	0.456
	*α*_*m*_	**0.224**	**0.076**	**0.078**	**0.079**
	λ	0.947	0.053	0.078	0.064
	*β*	1.335	0.865	0.376	0.255
	*β*_*m*_	−**0.260**	**0.140**	**0.082**	**0.048**
	*σ*_*b*_	0.885	0.115	0.165	0.060
Logistic regression true data	*α*	−1.072	0.728		
	*α*_*m*_	**0.188**	**0.112**		
Cox regression true data	*β*	1.996	0.204		
	*β*_*m*_	−**0.329**	**0.071**		
Logistic regression *σ*_*err*_ = 0.5	*α*	−1.047	0.75253		
	*α*_*m*_	**0.180**	**0.120**		
Cox regression *σ*_*err*_ = 0.5	*β*	2.019	0.181		
	*β*_*m*_	−**0.297**	**0.103**		
Logistic regression *σ*_*err*_ = 0.75	*α*	−0.965	0.835		
	*α*_*m*_	**0.166**	**0.134**		
Cox regression *σ*_*err*_ = 0.75	*β*	1.992	0.208		
	*β*_*m*_	−**0.221**	**0.179**		
Logistic regression *σ*_*err*_ = 1.0	*α*	−0.926	0.874		
	*α*_*m*_	**0.150**	**0.150**		
Cox regression *σ*_*err*_ = 1.0	*β*	1.973	0.227		
	*β*_*m*_	−**0.177**	**0.223**		

Bold value represents the TMB effect.

According to Table 1, the regression parameter estimates for the two function components perform reasonably well for a variety of measurement error conditions. In the absence of measurement errors, the joint model outperforms ordinary regression models in calculating regression coefficients because it more precisely reflects the potential connections between several endpoints. When considering different levels of measurement errors, the performance of the estimator based on corrected score was significantly superior to that of the naive estimator and only marginally poorer than that of the true-data estimator. Clearly, the performance of the naive estimator deteriorates with increasing error magnitude, which further suggests that the measurement error introduces a more significant bias effect on the parameter estimates. Overall, the results of the simulation experiments support the proposed joint multi-endpoint model and the iterative numerical estimation procedure, as well as the applicability of the associated random effects. Additionally, comparing SE and SD, the precision of the stated standard errors is generally satisfactory. The biases of the joint assessments compared to the standard separate regressions emphasize that the dependence among clinical endpoints could be an important and non-negligible confounder in analyzing the factors determining the treatment effect.

**FIGURE 2:**
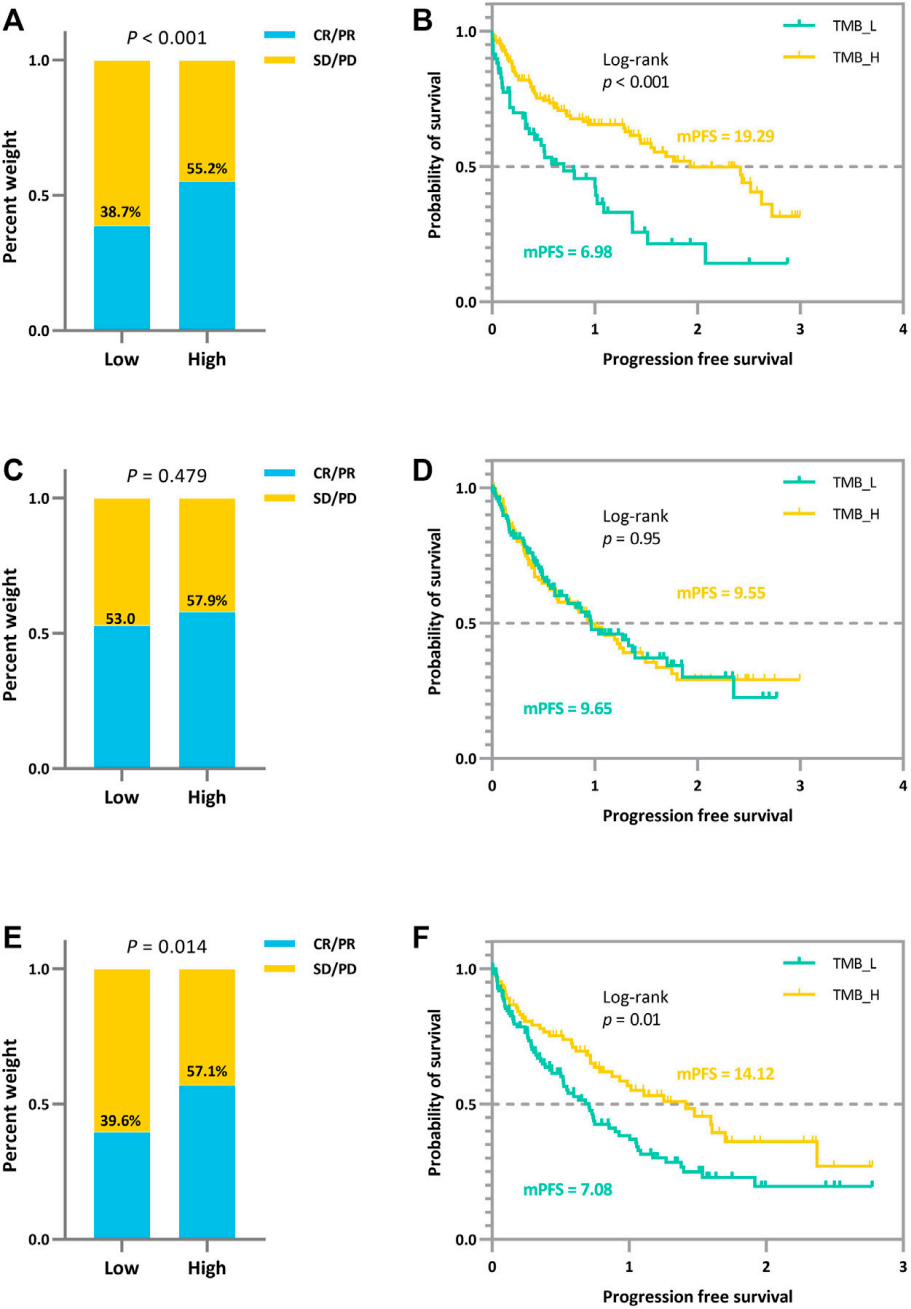
Efficacy comparison of patients grouped based on different TMB thresholds. **(A) (B)** Comparison of ORR and survival curves based on the threshold derived from the joint statistical inference with TMB actual values. **(C) (D)** Comparison of ORR and survival curves based on median TMB observations. **(E) (F)** Comparison of ORR and survival curves based on the threshold derived from the joint-correction statistical inference with TMB observed values.

To further exhibit the disturbance of measurement errors on TMB thresholds and the stability of our proposed joint model, we additionally simulated the comparison of efficacy grouped by different TMB thresholds. We simulated the prognosis of a cohort of patients based on the assumption that there is a positive correlation between actual TMB levels and a favorable immunotherapy prognosis, with coefficients set exactly as above. The variance fluctuation of TMB measurement error was set to 1.0. We derive the different thresholds for classifying patients and comparing their efficacy based on the joint statistical inference with the TMB actual values, the quantile method with TMB observations, and the joint-correction statistical inference with TMB observations. The outcomes of the comparison are depicted in Figure 2. We can clearly observe that the discrepancies between the efficacies of different groups are minimized or even reversed (Figure 2C,D) when the patients were classified directly using quartiles in the presence of measurement errors. Contrary to the clinical theory that the higher TMB, the more antitumor immunogenic the patient, patients in the TMB-low subgroup demonstrated greater therapeutic benefit in terms of tumor remission and progression-free survival than those in the TMB-high subgroup. The confounding effect of the measurement errors would dilute the actual link between TMB levels and immunotherapy clinical efficacy (Figures 2A,B), preventing appropriate screening for superior patient populations. In contrast, the bias effect due to measurement errors is reduced when we use the joint model as well as the correction estimation procedure. As shown in Figure 2E,F, the TMB threshold determination based on our proposed method ensures both the validity and a certain degree of error tolerance.

### Patient Cohort Characteristics

**FIGURE 3:**
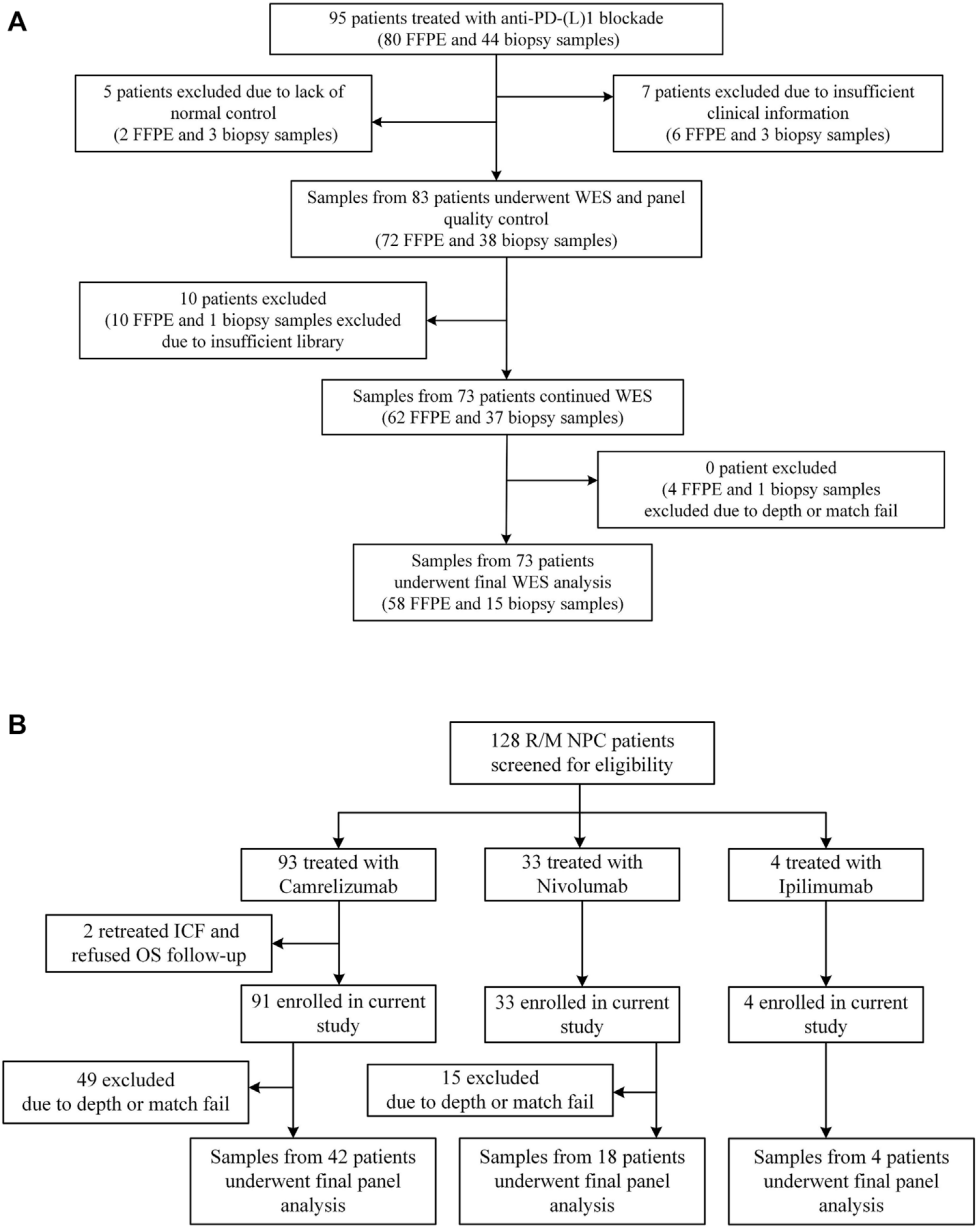
Patient samples included in the final analysis. **(A)** Flowchart for NSCLC sample inclusions. Among the 95 patients who underwent anti-PD-(L) 1 therapies and had available FFPE and/or biopsy tumor samples, we performed WES on samples from 73 patients. **(B)** Flowchart for NPC sample inclusions. Among the 128 patients who underwent anti-PD-(L) 1 or anti-CTLA-4 therapies, we performed targeted NGS on samples from 64 patients.

Sun Yat-sen University Cancer Center recruited 95 NSCLC patients who received anti-PD-(L)1 monotherapy between December 2015 and August 2017, with data collected until January 2019. The study design has already been published (Fang et al., 2019). Between March 2016 and January 2018, R/M NPC patients have enrolled in two single-arm phase I trials (NCT02721589 and NCT02593786), where 128 patients were screened for eligibility. The dose escalation and expansion phases of the study were previously reported (Fang et al., 2018; Ma et al., 2019). Eligible patients aged from 18 to 70 years had histologically or cytologically confirmed locally advanced or metastatic NSCLC or NPC, had an Eastern Cooperative Oncology Group (ECOG) performance-status score of 0 or 1 (on a 5-point scale, with higher numbers indicating greater disability), had at least one measurable lesion according to the Response Evaluation Criteria in Solid Tumors (RECIST version 1.1 (Eisenhauer et al., 2009)), and had failed at least one prior line of systemic therapy. Figure 3 and Supplementary Table S1 depict the distribution of patients’ treatments. Radiographic tumor assessments were taken at the start of the study and every 6 weeks thereafter. The proportion of patients with complete response (CR) and partial response (PR) was known as the ORR. The time from the initial dose until PD or any-cause death was referred to as progression-free survival (PFS). Censored data documented the last radiographic assessment before cut-off, follow-up loss, or treatment change. Overall survival (OS) was defined as the time from the first dosage to death, and patients who remained alive were censored at the date of their last follow-up. The Sun Yat-sen University Cancer Center’s Ethical Review Committee approved this study, which was carried out in conformity with the Declaration of Helsinki. Each patient signed the written informed consent.

At Sun Yat-sen University Cancer Center, 95 Chinese patients with NSCLC were treated with anti-PD-(L)1 monotherapies, with 73 patients being included in the final analysis with evaluable radiological results. Concurrently, 128 patients with R/M NPC who had received anti-PD-(L)1 monotherapies were retrospectively investigated, of whom 64 patients were being screened for the final analysis based on sequencing quality and follow-up completeness. When both FFPE and biopsy samples were available for the patient, the FFPE sample was used in the analysis, given the limited intra-tumoral heterogeneity represented by a single biopsy sample. The study design and clinical characteristics of this cohort are summarized in Figure 3 and Table 2 with details in Supplementary Table S1. For lung cancer, 60% of the patients had adenocarcinoma, followed by squamous carcinoma (32%). Almost all patients (99%) were stage IV at diagnosis; the median age of patients with NSCLC and NPC at the treatment initiation was 55 and 46 years, respectively. 49% of the NSCLC patients and 25% NPC patients had a smoking history and more males in both cohorts (70% vs. 30% for NSCLC, 80% vs. 20% for NPC). ORR of the study cohorts was 19% and 12%, and the median progression-free survival (mPFS) was 91 days for lung cancer and 67.5 days for NPC. No difference in PFS was observed among the different immune agents.

**TABLE 2:** Baseline clinical characteristics for NSCLC patients and NPC patients.

Characteristic for NSCLC patients	All patients (*N* = 73)
Median age (range)	55 (28–73)
Sex—No. (%)	
Male	51 (70%)
Female	22 (30%)
ORR—No. (%)	
CR/PR	14 (19%)
SD	20 (27%)
PD	39 (54%)
Stage—No. (%)	
III	1 (1%)
IV	72 (99%)
Immunotherapy—No. (%)	
Anti-PD-1	68 (93%)
Anti-PD-L1	5 (7%)
Smoking status—No. (%)	
Current or former smoker	36 (49%)
Never smoker	47 (51%)
Pathological type—No. (%)	
Adenocarcinoma	44 (60%)
Squamous carcinoma	23 (32%)
Others	6 (8%)
**Characteristic for NPC patients**	**All patients (*N* = 64)**
Median age (range)	46 (23–73)
Sex—No. (%)	
Male	51 (80%)
Female	13 (20%)
ORR—No. (%)	
CR/PR	8 (12%)
SD	19 (30%)
PD	37 (58%)
Stage—No. (%)	
IV	64 (100%)
Immunotherapy—No. (%)	
Camrelizumab	42 (66%)
Nivolumab	18 (28%)
Ipilimumab	4 (6%)
Smoking status—No. (%)	
Current or former smoker	16 (25%)
Never smoker	48 (75%)
Therapy line—No. (%)	
2	15 (23%)
>2	42 (66%)
NA	7 (11%)

In addition to the SYSUCC NSCLC cohort and NPC cohort described above, external cohorts of 943 patients from the public literatures treated with ICI are compiled in Supplementary Table S2, encompassing 453 melanoma patients (Snyder et al., 2014; Van Allen et al., 2015; Hugo et al., 2016; Goodman et al., 2017), 407 NSCLC patients (Goodman et al., 2017; Hellmann et al., 2018a; Miao et al., 2018; Rizvi et al., 2018), 56 RCC (Wood et al., 2020), and 27 bladder (Miao et al., 2018), along with treatment modality and outcome analyzed. The mutation callings are derived from three sequencing platforms (WES, F1CDx, and MSK-IMPACT). F1CDx and MSK-IMPACT are NGS targeted panel being authorized by the FDA as practical diagnostic assays. Table 3 summarizes the sequencing methodology and varied TMB thresholds employed in the gathered research.

**TABLE 3:** Patient cohorts from the published literatures.

Cancer type	Num.	Sequencing platform	TMB threshold	Case
NSCLC	35	F1CDx	≥20 mut/Mb	Goodman et al. (2017)
	57	WES	No definition	Miao et al. (2018)
	75	WES	Median	Hellmann et al. (2018a)
	240	MSK-IMPACT	Median	Rizvi et al. (2018)
Melanoma	37	WES	Top third	Hugo et al. (2016)
	52	F1CDx	≥20 mut/Mb	Goodman et al. (2017)
	64	WES	≥100 mut/Mb	Snyder et al. (2014)
	105	WES	≥100 mut/Mb	Van Allen et al. (2015)
	195 (58)^a^	WES(MC3)	75th percentiles	Wood et al. (2020)
RCC	56	WES(MC3)	75th percentiles	Wood et al. (2020)
Bladder	27	WES	No definition	Miao et al. (2018)

a Wood2020 study is a pooling meta-analysis on several existing datasets, where 58 patients of the 195 were patients not included in the above studies.

### Joint Model Prompts a Comprehensive and Robust TMB Subgrouping

The multi-endpoint joint analysis used to locate TMB thresholds is superior to the previous studies as it provides a more comprehensive analysis of patient clinical information. Based on the co-analyzed labels, it can give an overall picture of disease efficacy. Based on these compound indices to establish ROC curves to handle true- and false-positive rates in the classification, we selected a TMB threshold from clinically meaningful values to group patients in the experiment and validation sets.

**FIGURE 4:**
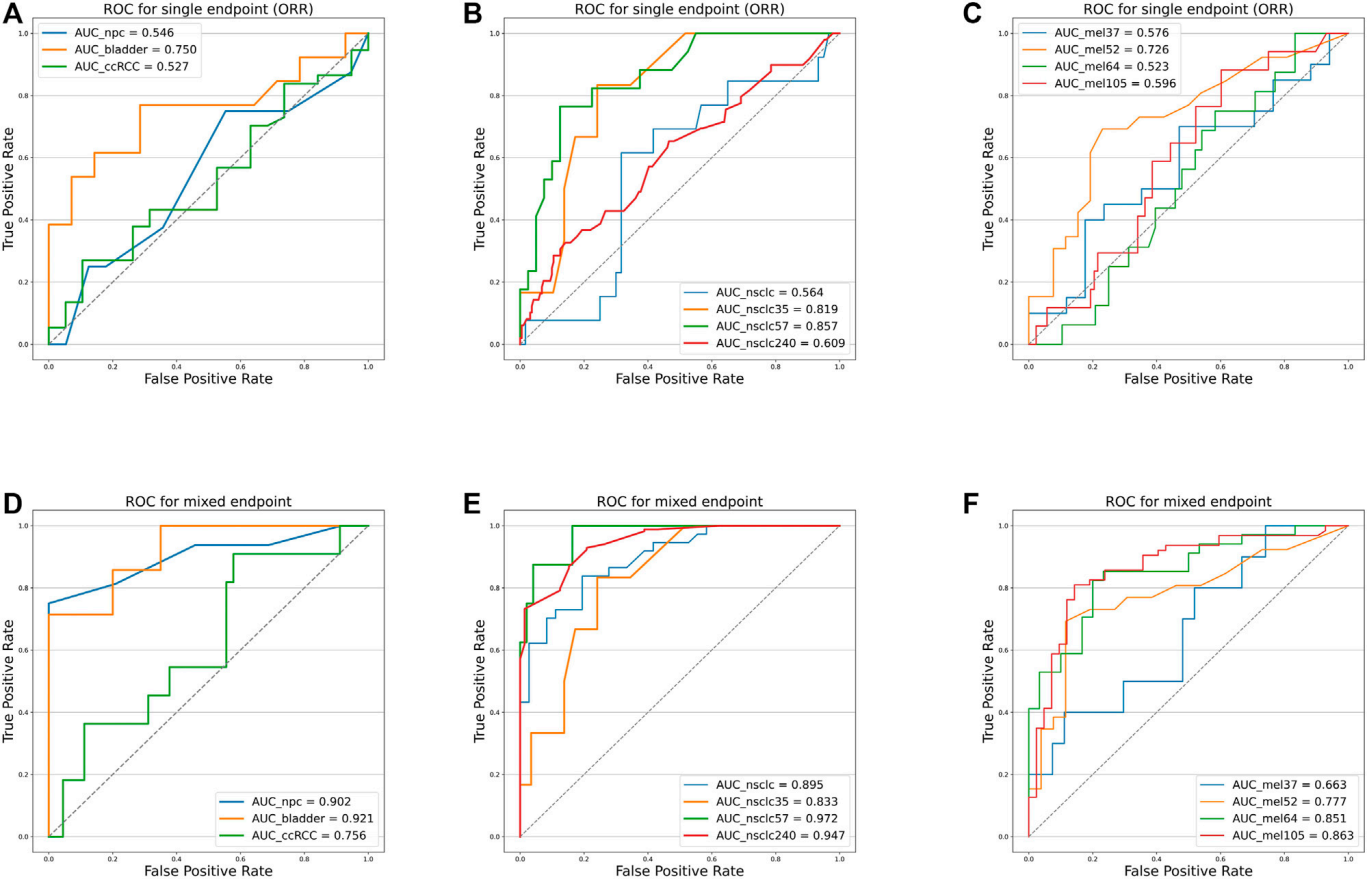
Receiver operating characteristic curves of the predictive capacity of prognosis label for two experiment cohorts and validation cohorts, depicting the true-positive rate (sensitivity, *y*-axis) and false-positive rate (1-specificity, *x*-axis) for the metric across all possible TMB thresholds. The corresponding area under the curve (AUC) is illustrated in the figure legends. **(A)** ROC curves for NPC (panel) experiment cohort, bladder cohort, and RCC cohort based on ORR labels alone. **(B)** ROC curves for NSCLC (WES) experiment cohort, NSCLC_35 cohort, NSCLC_57 cohort, and NSCLC_240 cohort based on ORR labels alone. **(C)** ROC curves for Mel_37 cohort, Mel_52 cohort, Mel_64 cohort, and Mel_105 cohort based on ORR labels alone. **(D)** ROC curves for NPC (panel) experiment cohort, bladder cohort, and RCC cohort based on the mixed-endpoint labels. **(E)** ROC curves for NSCLC (WES) experiment cohort, NSCLC_35 cohort, NSCLC_57 cohort, and NSCLC_240 cohort based on the mixed-endpoint labels. **(F)** ROC curves for Mel_37 cohort, Mel_52 cohort, Mel_64 cohort, and Mel_105 cohort based on the mixed-endpoint labels.

As shown in Figure 4 and Table 4, we can discern that the ROC curves based on the mixed-endpoint joint labels generally had higher AUCs in either the experiment or validation groups, with an average improvement of about 0.2 over those based on ORR labels alone, and the range of confidence intervals likewise supports this conclusion. More importantly, all the AUCs established on the proposed indices exceeded 0.6, ranges from 0.663 to 0.972, reflecting our model’s more robust discrimination capabilities. For comparison, as for the ROCs based on original ORR labels, despite the classification ability varying among cancer types, the ROCs in most cases showed more inferiority, with half of the cases only marginally exceeding 0.5 not reaching 0.6, even equivalent to random chance. The results in Figure 4 and Table 4 fully demonstrate that the subgrouping of TMB under the joint modeling of multiple endpoints is significantly improved compared to the existing subgrouping based on the ORR single label. We attribute this phenomenon to a proportion of the patients with opposing effects on the two rubrics present in these cases. Although high TMB was reported associated with ICI treatment improvement in terms of overall trends, the status of a single indicator alone is not fully representative of the patient’s actual matter. This is why the ROC curves established based on only a single endpoint have such poor performance. Integrating patients’ multi-dimensional information and joint modeling mixed-endpoints can prompt a more comprehensive stratification of TMB. Our approach could provide clinicians with a full assessment of efficacy, resulting in a comprehensive determination of the TMB screening threshold for superior patients.

**TABLE 4:** AUC comparison. The table reports the area under the curve (AUC), as well as the corresponding 0.95 confidence interval, for each metric (columns) applied to a different cancer cohort (rows). Bold-faced values indicate the best value for each cancer cohort.

Experiment cohort	AUC based on ORR	0.95 CI	AUC based on joint model	0.95 CI
NPC	0.546	0.321–0.77	**0.902**	**0.793–1.000**
NSCLC	0.564	0.398–0.730	**0.895**	**0.826–0.964**
**Validation cohort**	**AUC based on ORR**	**0.95 CI**	**AUC based on joint model**	**0.95 CI**
Bladder	0.750	0.554–0.946	**0.921**	**0.807–1.000**
RCC	0.527	0.370–0.684	**0.756**	**0.594–0.918**
NSCLC_35	0.819	0.668–0.970	**0.833**	**0.683–0.983**
NSCLC_57	0.857	0.756–0.959	**0.972**	**0.928–1.000**
NSCLC_240	0.609	0.517–0.701	**0.947**	**0.922–0.973**
Mel_37	0.576	0.389–0.764	**0.663**	**0.467–0.859**
Mel_52	0.726	0.585–0.866	**0.777**	**0.646–0.909**
Mel_64	0.523	0.375–0.671	**0.851**	**0.757–0.943**
Mel_105	0.596	0.466–0.726	**0.863**	**0.789–0.937**

**FIGURE 5:**
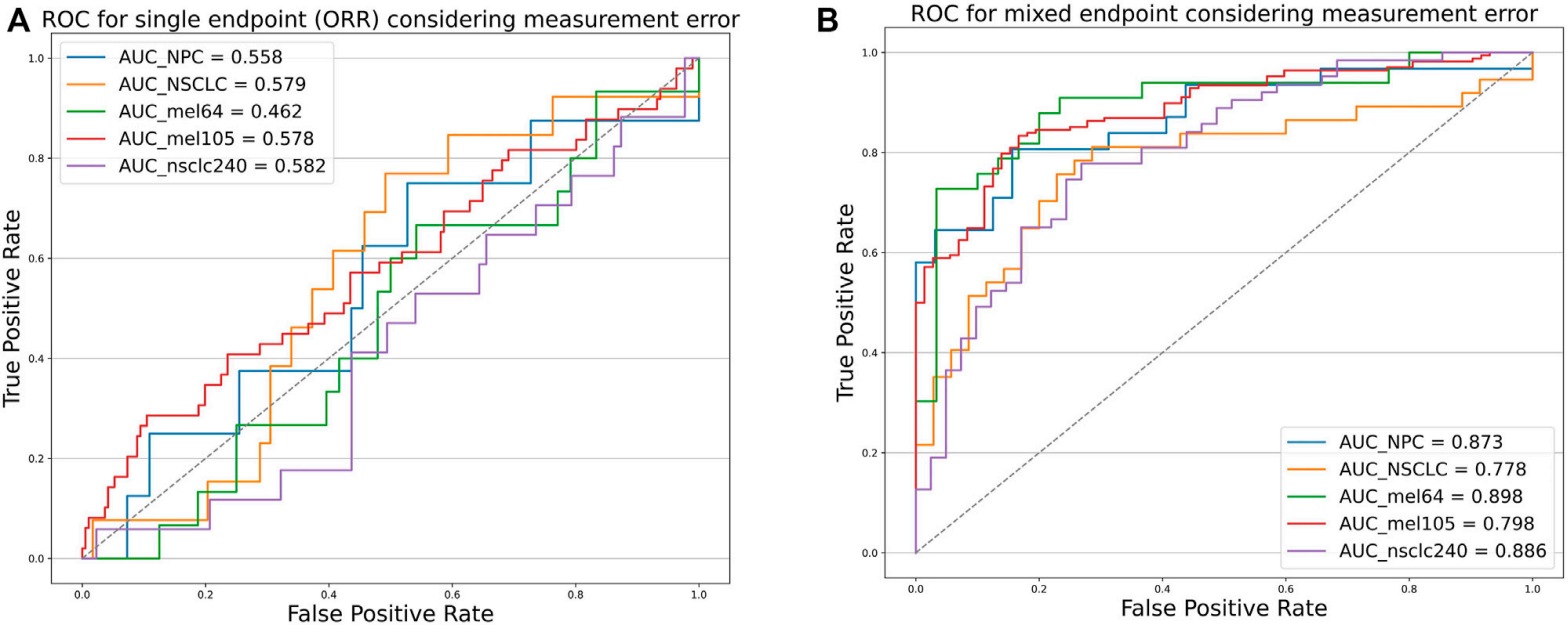
Receiver operating characteristic curves of the predictive capacity of prognosis label for two experiment cohorts and validation cohorts, depicting the true-positive rate (sensitivity, *y*-axis) and false-positive rate (1-specificity, *x*-axis) for the metric across all possible TMB thresholds considering measurement errors. The corresponding area under the curve (AUC) is illustrated in the figure legends. **(A)** ROC curves for NPC (Panel), NSCLC (WES) experiment cohort, Mel_64 cohort, Mel_105 cohort, and NSCLC_240 cohort based on ORR labels considering TMB measurement errors. **(B)** ROC curves for NPC (panel), NSCLC (WES) experiment cohort, Mel_64 cohort, Mel_105 cohort, and NSCLC_240 cohort based on the mixed-endpoint labels considering TMB measurement errors.

To verify that our proposed threshold delineation method for TMB remains valid and robust under the perturbation of measurement errors, we added 10%–20% artificial noise according to the actual TMB level. Given the small number of patients in some cases, which are over-sensitive to data noise, we selected several groups of cases with more patients for analysis. The results are shown in Figure 5 and Table 5. Under the perturbation of artificial noise, the AUC of each group showed mostly a slight decrease compared to the error-free cases. However, the ROC curves based on our proposed joint labels still maintain a high AUC, which is about 0.3 higher on average than the ROC curves based on ORR labels only. These results demonstrate the high error tolerance of our proposed joint model.

**TABLE 5:** AUC comparison. The table reports the area under the curve (AUC), as well as the corresponding 0.95 confidence interval, for each metric (columns) applied to a different cancer cohort (rows). Bold-faced values indicate the best value for each cancer cohort.

Experiment cohort	AUC based on ORR	0.95 CI	AUC based on joint model	0.95 CI
NPC	0.558	0.341–0.775	**0.873**	**0.783–0.963**
NSCLC	0.579	0.421–0.737	**0.778**	**0.665–0.890**
**Validation cohort**	**AUC based on ORR**	**0.95 CI**	**AUC based on joint model**	**0.95 CI**
NSCLC_240	0.582	0.487–0.677	**0.886**	**0.845–0.928**
Mel_64	0.462	0.307–0.617	**0.898**	**0.817–0.979**
Mel_105	0.578	0.436–0.720	**0.798**	**0.712–0.884**

### Joint Analysis Prompts a Significant and Error-Tolerant Patient Subgrouping

**FIGURE 6:**
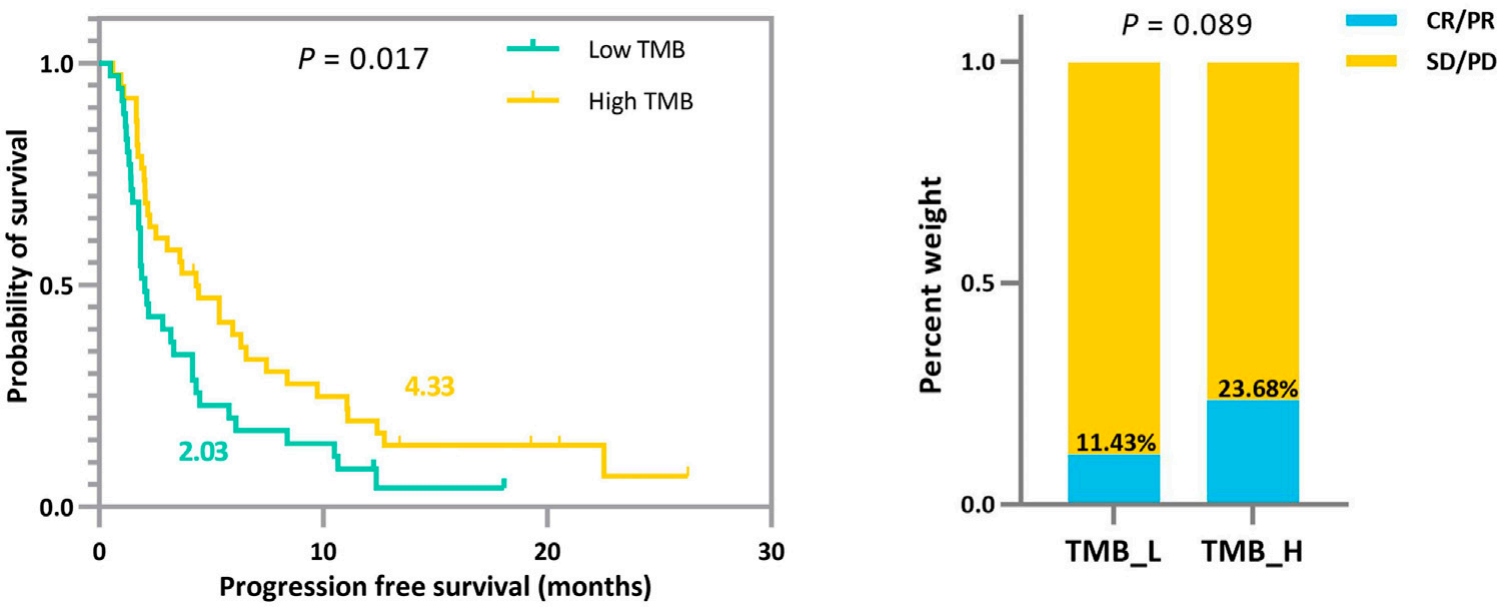
Survival curves and ORR comparison between experimental NSCLC patients (*n* = 73) with low and high TMB. Improved progression-free survival (PFS) and a trend toward increased objective response rate (ORR) are observed in patients with high TMB.

In addition to the strengths shown in the ROC curves, based on the derived TMB thresholds, we can classify experimental NSCLC patients into two groups with apparently stratified efficacy. The effect of the dichotomy is shown in Figure 6, where we can notice a significant difference between patients in *TMB_Low* and *TMB_High* in terms of immunotherapy benefit (*p*-values = 0.017 and 0.089). The grouping results on the other cohorts can be seen in Supplementary Figures.

To demonstrate that the TMB thresholds derived from our proposed joint model can significantly separate the treatment effects of patients receiving immunotherapy, we statistically compared the patient outcomes obtained based on our thresholds with those obtained from different quartiles (median, upper tertile, upper quartile) using the log-rank test and the two-sided Mann–Whitney U test. As shown by the results in Table 6, our model-derived TMB thresholds performed satisfactorily and consistently for cohort patient segmentation. This predominance is mainly reflected in the *p*-values of the statistical tests, which are essentially the lowest among all division scenarios under the threshold division based on the joint model, indicating that the proposed model is predominate. NSCLC_35 and NSCLC_240 were the only two situations in which the *p*-values performed marginally worse than the quantile divisions. Similarly, five groups of patients were selected to validate the stability of the proposed model in the face of the TMB measurement error. As shown by the results in Table 7, our proposed threshold delineation method for TMB remained efficient and robust under perturbation of measurement error.

**TABLE 6:** Immunotherapy mPFS or mOS and response probability based on different tumor mutation burden (TMB) thresholds for non-small-cell lung cancer (NSCLC), nasopharyngeal carcinoma (NPC), bladder, renal cell carcinoma (RCC), and melanoma. *p* values are reported by log-rank test and the two-sided Mann–Whitney U test.

Case	Threshold	mPFS or mOS (months) for TMB_L	mPFS or mOS for (months) TMB_H	*p*-value	Response Prob for TMB_L (%)	Response Prob for TMB_H (%)	*p*-value
NSCLC	**Joint model**	**2.03**	**4.33**	**0.017**	**11.43**	**23.68**	**0.089**
	Median	2.03	4.33	0.028	11.11	24.32	0.073
	Top third	2.17	5.37	0.023	16.33	20.83	0.323
	75th	2.27	4.33	0.713	20.75	10.00	0.146
NPC	**Joint model**	**1.77**	**2.57**	**0.791**	**7.40**	**16.22**	**0.151**
	Median	1.77	2.57	0.791	7.40	16.22	0.151
	Top third	1.93	2.57	0.755	12.20	13.04	0.466
	75th	1.93	2.57	0.755	12.20	13.04	0.466
Bladder	**Joint model**	**16.71**	**16.55**	**0.243**	**36.36**	**100.00**	**0.009**
	Median	16.71	16.55	0.535	23.08	71.43	0.012
	Top third	16.71	16.12	0.806	33.33	77.78	0.038
	75th	16.71	16.12	0.437	35.00	85.71	0.023
RCC	**Joint model**	**5.70**	**2.70**	**0.335**	**62.79**	**76.92**	**0.178**
	Median	6.80	3.60	0.955	67.86	64.29	0.394
	Top third	5.77	3.97	0.982	62.16	73.68	0.199
	75th	5.60	4.30	0.808	64.29	71.43	0.318
NSCLC_35	**Joint model**	**1.80**	**4.00**	**0.040**	**4.35**	**41.67**	**0.003**
	Median	2.00	3.20	0.137	0.00	28.57	0.016
	Top third	1.80	4.00	0.024	4.35	41.67	0.003
	75th	1.80	4.00	0.015	7.69	44.44	0.007
NSCLC_57	**Joint model**	**10.39**	**14.61**	**<0.001**	**12.50**	**70.59**	**<0.001**
	Median	10.39	14.61	0.001	32.14	41.38	<0.001
	Top third	10.39	14.61	**<0.001**	44.74	21.05	<0.001
	75th	10.39	14.61	0.002	40.47	26.67	<0.001
NSCLC_75	**Joint model**	**3.78**	**22.14**	**0.006**	**12.20**	**55.90**	**<0.001**
	Median	3.78	8.12	0.012	13.51	50.00	0.002
	Top third	3.94	22.14	0.003	20.00	56.00	0.001
	75th	5.10	23.0	0.019	23.21	57.89	0.004
NSCLC_240	**Joint model**	**3.10**	**4.17**	**0.062**	**14.29**	**26.45**	**0.052**
	Median	3.10	4.17	0.062	14.29	26.45	0.052
	Top third	3.03	4.20	0.235	17.83	25.30	0.108
	75th	2.73	5.47	0.030	17.22	30.00	0.002
Mel_37	**Joint model**	**27.40**	**32.10**	**0.055**	**40.00**	**63.64**	**0.084**
	Median	27.40	31.2	0.044	50.00	57.89	0.324
	Top third	31.00	32.10	0.151	48.00	66.67	0.151
	75th	31.00	32.10	0.561	48.15	70.00	0.125
Mel_52	**Joint model**	**5.80**	**40.000**	**0.121**	**32.26**	**76.19**	**<0.001**
	Median	6.80	15.20	0.250	30.77	69.23	0.003
	Top third	7.90	15.20	0.554	40.00	70.59	0.021
	75th	9.20	40.00	0.927	43.59	69.23	0.058
Mel_64	**Joint model**	**18.51**	**94.60**	**0.037**	**22.22**	**26.09**	**0.379**
	Median	19.79	*inf*	0.933	21.88	28.12	0.286
	Top third	32.4	44.40	0.868	25.58	23.81	0.443
	75th	32.84	*inf*	0.636	25.00	25.00	0.500
Mel_105	**Joint model**	**2.80**	**3.00**	**0.200**	**12.90**	**20.93**	**0.083**
	Median	2.80	3.00	0.622	11.54	20.75	0.129
	Top third	2.80	3.00	0.851	17.14	14.29	0.484
	75th	2.80	3.30	0.606	15.39	18.52	0.237
Mel_195	**Joint model**	**3.73**	**6.06**	**0.607**	**29.07**	**38.89**	**0.077**
	Median	3.73	4.90	0.640	28.87	40.21	0.049
	Top third	4.63	3.80	0.730	34.88	33.85	0.444
	75th	5.10	3.33	0.090	35.86	30.61	0.253

Bold values represent the results of the proposed joint model.

**TABLE 7:** Immunotherapy mPFS or mOS and response probability based on different tumor mutation burden (TMB) thresholds with measurement errors for non-small-cell lung cancer (NSCLC), nasopharyngeal carcinoma (NPC), bladder, renal cell carcinoma (RCC), and melanoma. *p* values are reported by log-rank test and the two-sided Mann–Whitney U test.

Case	Threshold	mPFS or mOS (months) for TMB_L	mPFS or mOS for (months) TMB_H	*p*-value	Response Prob for TMB_L (%)	Response Prob for TMB_H (%)	*p*-value
NSCLC	**Joint model**	**2.03**	**4.33**	**0.022**	**9.375**	**24.39**	**0.100**
	Median	2.13	4.33	0.046	11.11	24.31	0.145
	Top third	2.17	4.43	0.023	14.12	22.95	0.171
	75th	2.17	4.43	0.010	16.55	20.00	0.522
NPC	**Joint model**	**1.77**	**2.57**	**0.543**	**6.25**	**18.75**	**0.137**
	Median	1.77	2.57	0.543	6.25	18.75	0.137
	Top third	1.93	2.57	0.970	9.33	16.98	0.200
	75th	1.93	2.57	0.927	10.57	15.94	0.282
NSCLC_240	**Joint model**	**2.9**	**4.2**	**0.016**	**13.39**	**26.56**	**0.011**
	Median	3.1	3.77	0.264	17.5	23.33	0.140
	Top third	3.07	4.17	0.061	17.5	24.5	0.094
	75th	3.07	4.27	0.013	17.61	25.38	0.023
Mel_64	**Joint model**	**18.51**	***inf***	**0.204**	**16.13**	**33.33**	**0.12**
	Median	18.51	*inf*	0.259	18.75	31.25	0.257
	Top third	31.2	*inf*	0.297	22.67	28.30	0.472
	75th	32.4	*inf*	0.262	23.58	27.54	0.546
Mel_105	**Joint model**	**2.7**	**3.3**	**0.019**	**4.65**	**24.19**	**0.007**
	Median	2.8	3.27	0.835	15.38	16.98	0.829
	Top third	2.8	3.27	0.693	15.57	17.05	0.777
	75th	2.8	3.27	0.584	15.5	17.39	0.662

## Discussion

Tumor mutation burden has recently become an area of interest, as high TMB is associated with improved response to ICI therapies. However, the threshold defining the TMB-high/TMB-positive patients is controversial in clinical, which is exacerbated by the presence of multiple evaluation metrics and TMB calculation errors. The existing TMB threshold-identifying approaches are merely based on a single endpoint, which may suffer from excessive information loss. TMB metric, as a predictive marker, is closely associated with both of the two types of clinical endpoints (ORR and TTE), where the effect in two endpoints may be of different magnitude or even point in different directions. Herein, we report a generalized framework for comprehensively determining the positivity TMB thresholds based on a mixed-endpoint joint model and an iterative numerical estimation procedure considering measurement errors. In our joint model, we choose the Weibull–Cox proportional hazard model for the TTE endpoint. Although the baseline risk *h*_0_(*t*) in standard survival analysis usually be left unspecified, such as the advantageous partial likelihood method. However, within the joint modeling framework, it turns out that following such a route may lead to an underestimation of the standard errors of the parameter estimates (Hsieh et al., 2006). Thus, we recommend choosing an explicit definition of *h*_0_(*t*) based on the dataset characteristics, corresponding to a parametric distribution. The Weibull, the log–normal, and the Gamma distributions are typically employed in the survival analysis context. By analyzing the progression-free survival of patients receiving immunotherapy, we found that the trend of their baseline cumulative hazard distribution was consistent with the Weibull distribution with a scale parameter equal to 1 (see in Figure 1), so the Weibull–Cox proportional hazard model was employed in this article. Our joint model sheds new light on the tumor mutation burden stratification based on a multi-endpoint assessment of immunotherapy benefits, suggesting more comprehensive and robust TMB-positive thresholds for clinical physicians. Attending physicians should make treatment recommendations based on patients’ multi-dimensional information.

## Conclusion

The existing statistical methods for determining TMB thresholds are based on a single clinical endpoint while ignoring the difference between the true and observed TMB values. Our study considers TMB measurement error and integrates multifaceted clinical efficacy to optimize TMB thresholds. We report a multi-endpoint joint model as a generalized method for inferring TMB thresholds that facilitates consistent statistical inference using an iterative numerical estimation procedure considering mis-specified TMB. Our simulation results show that the proposed model maintains higher accuracy and stability than standard regressions, in terms of both parameter estimation and threshold determination. To validate the feasibility of the proposed thresholds, we pooled a cohort of 73 patients with non-small-cell lung cancer and 64 patients with nasopharyngeal carcinoma treated with anti-PD-(L)1, as well as a validation cohort of 943 patients for retrospective analysis. From the simulation and experimental results, we reasonably conclude that 1) our proposed joint model with the parameter estimation procedure can more robustly assess patient efficacy even under the interference of measurement error in TMB. 2) Integrating patients’ multi-dimensional information to employ multi-endpoint efficacy analysis can prompt a more comprehensive TMB subgrouping. 3) The TMB-positive threshold derived from multi-endpoint joint analysis can classify patients into two groups with more apparently stratified efficacy. Our model is applicable to clinical multiple endpoint data and can better assist physicians in their clinical decisions.

## Data Availability

The datasets presented in this study can be found in online repositories. The names of the repository/repositories and accession number(s) can be found in the article/Supplementary Material.
