# Supplementary figures and images for: A Joint Model Considering Measurement Errors for Optimally Identifying Tumor Mutation Burden Threshold
Source: Front Genet. 2022 Aug 4;13:915839. doi: 10.3389/fgene.2022.915839 (PMC9386083; doi:10.3389/fgene.2022.915839)

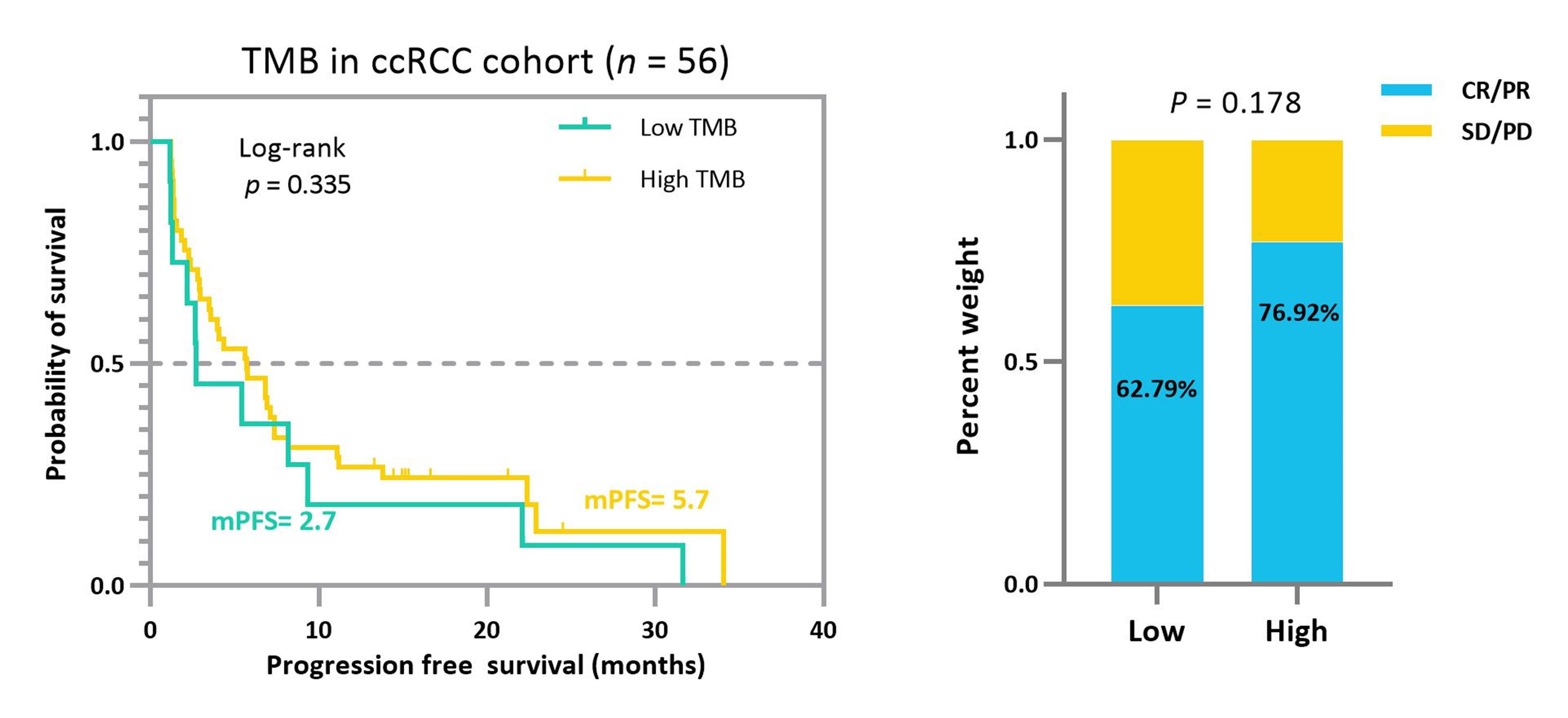

Supplement: Supplementary file 2 [file Image3.JPEG]

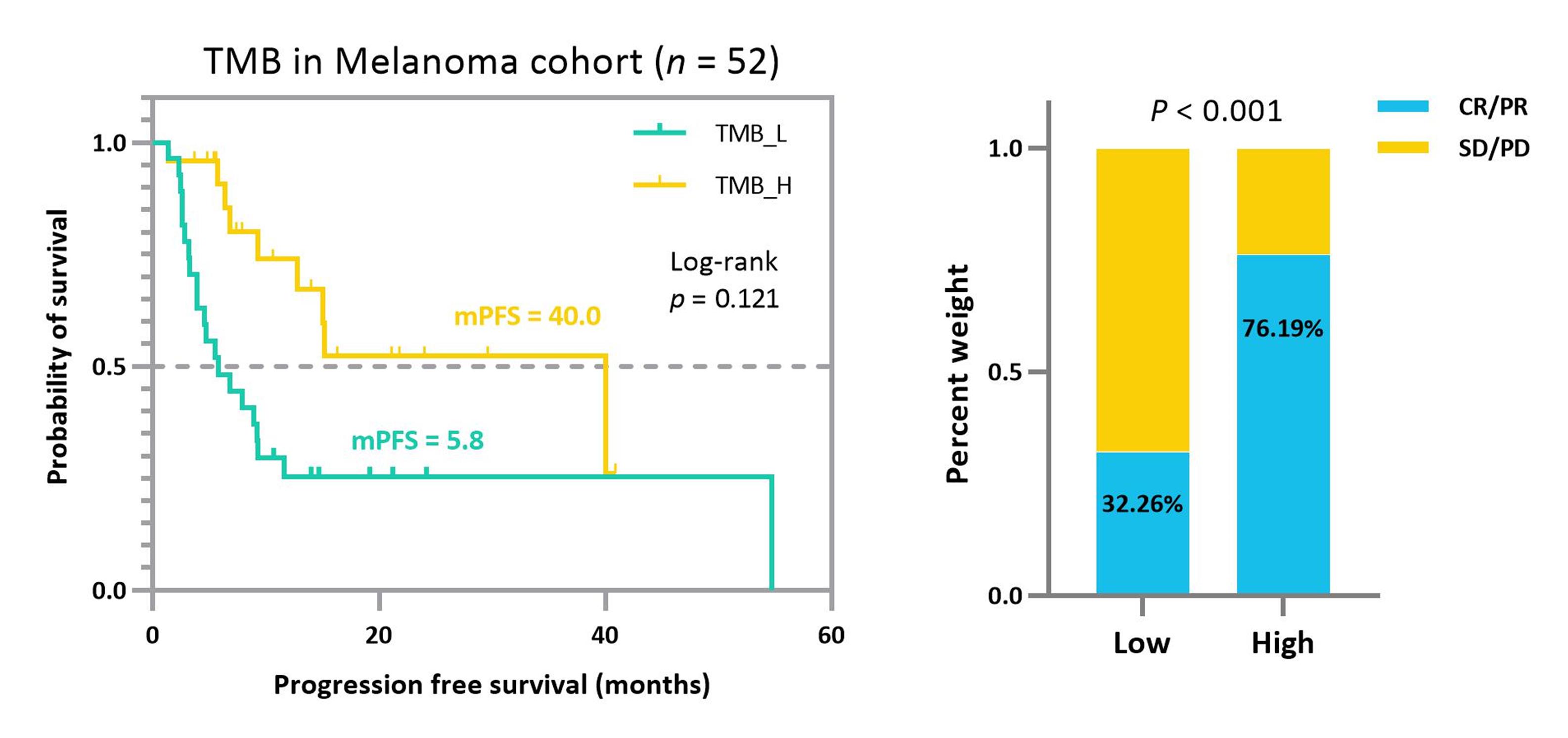

Supplement: Supplementary file 3 [file Image9.JPEG]

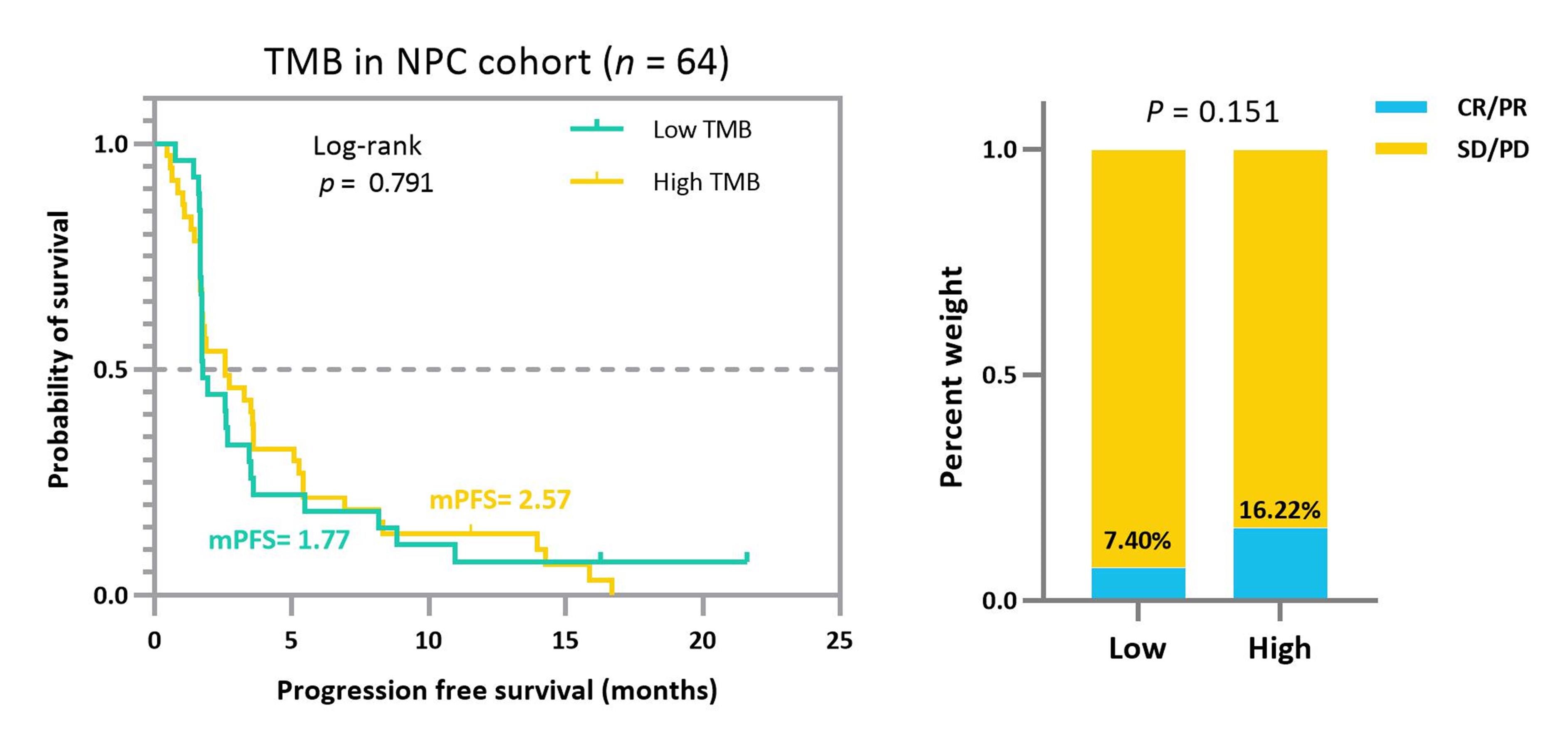

Supplement: Supplementary file 4 [file Image1.JPEG]

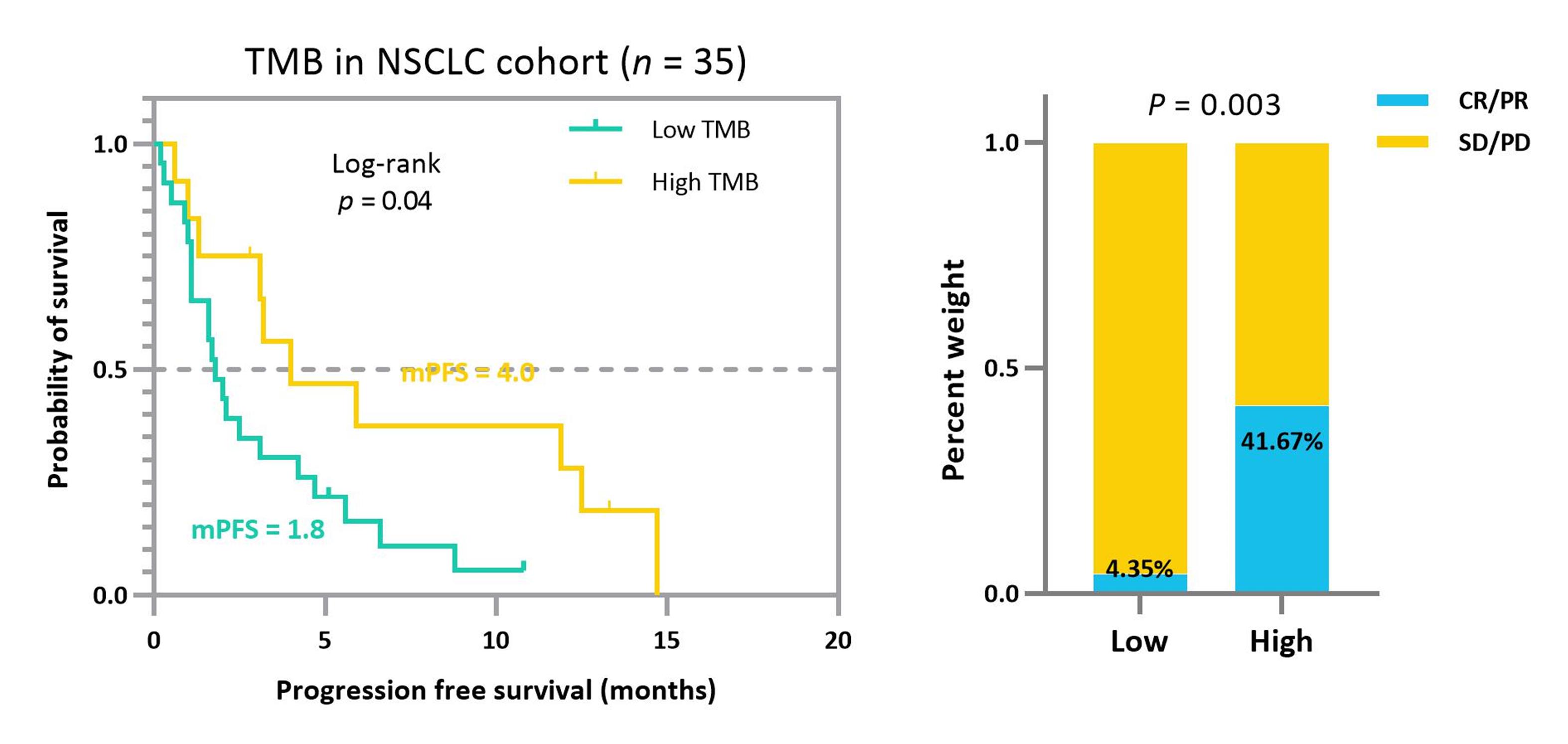

Supplement: Supplementary file 5 [file Image4.JPEG]

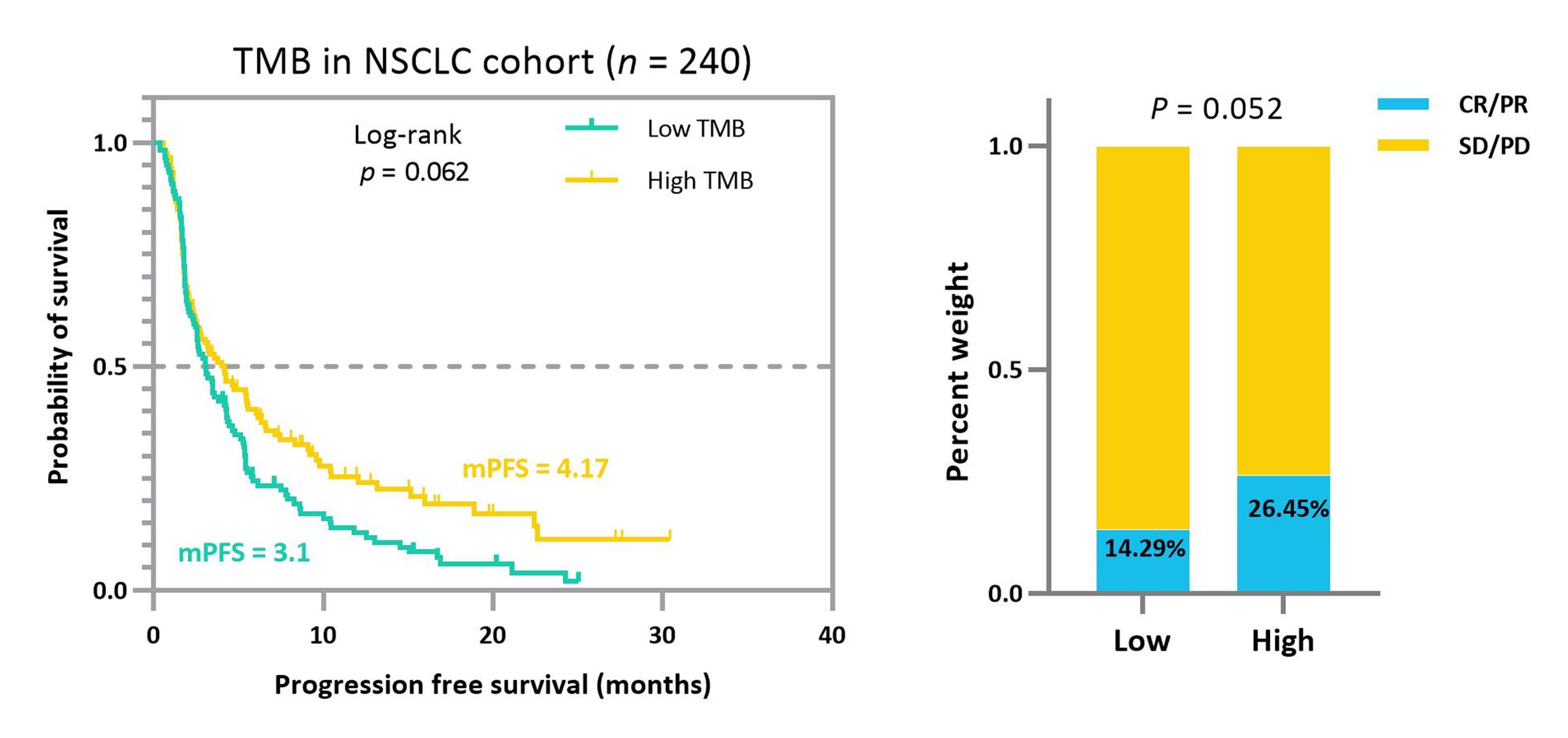

Supplement: Supplementary file 6 [file Image7.JPEG]

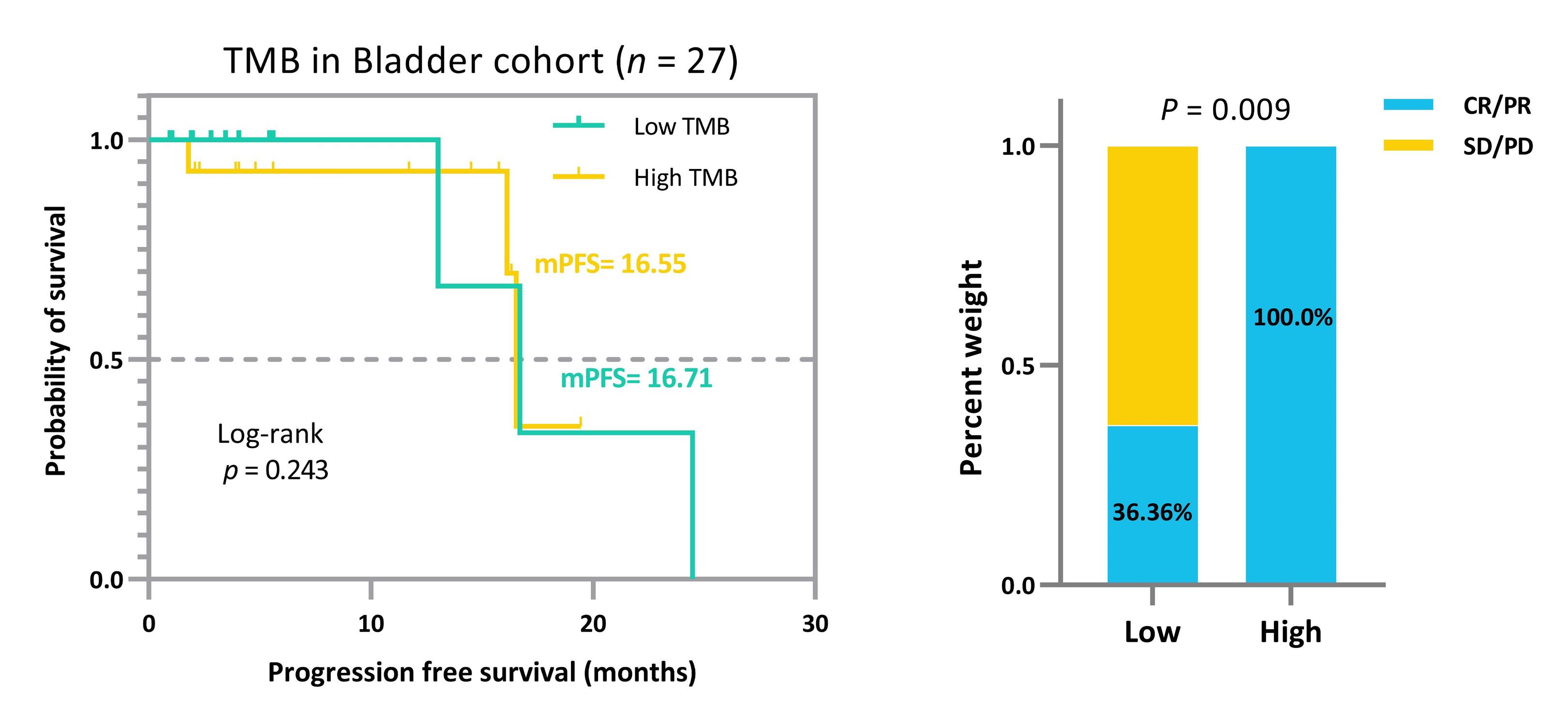

Supplement: Supplementary file 7 [file Image2.JPEG]

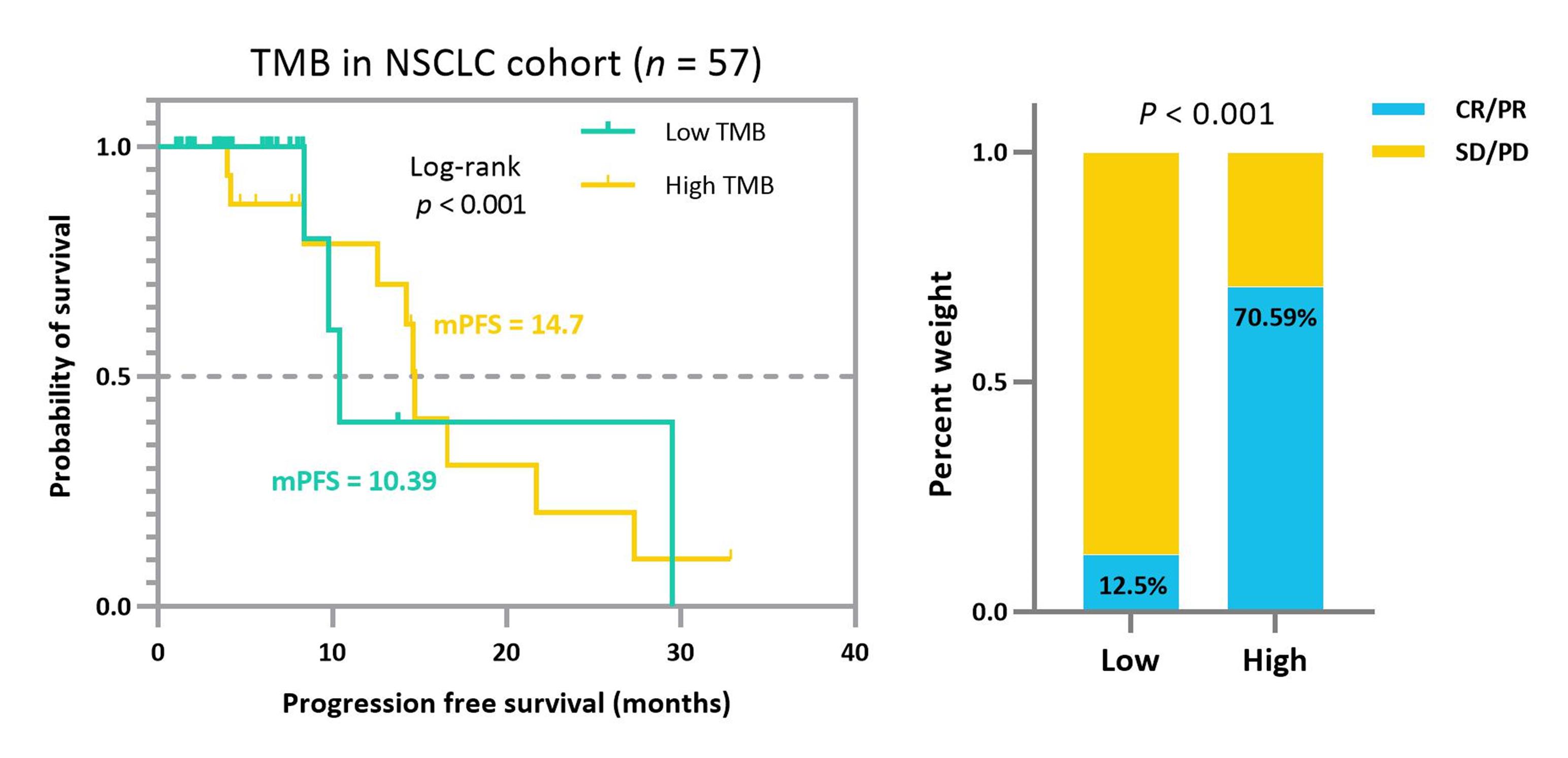

Supplement: Supplementary file 8 [file Image5.JPEG]

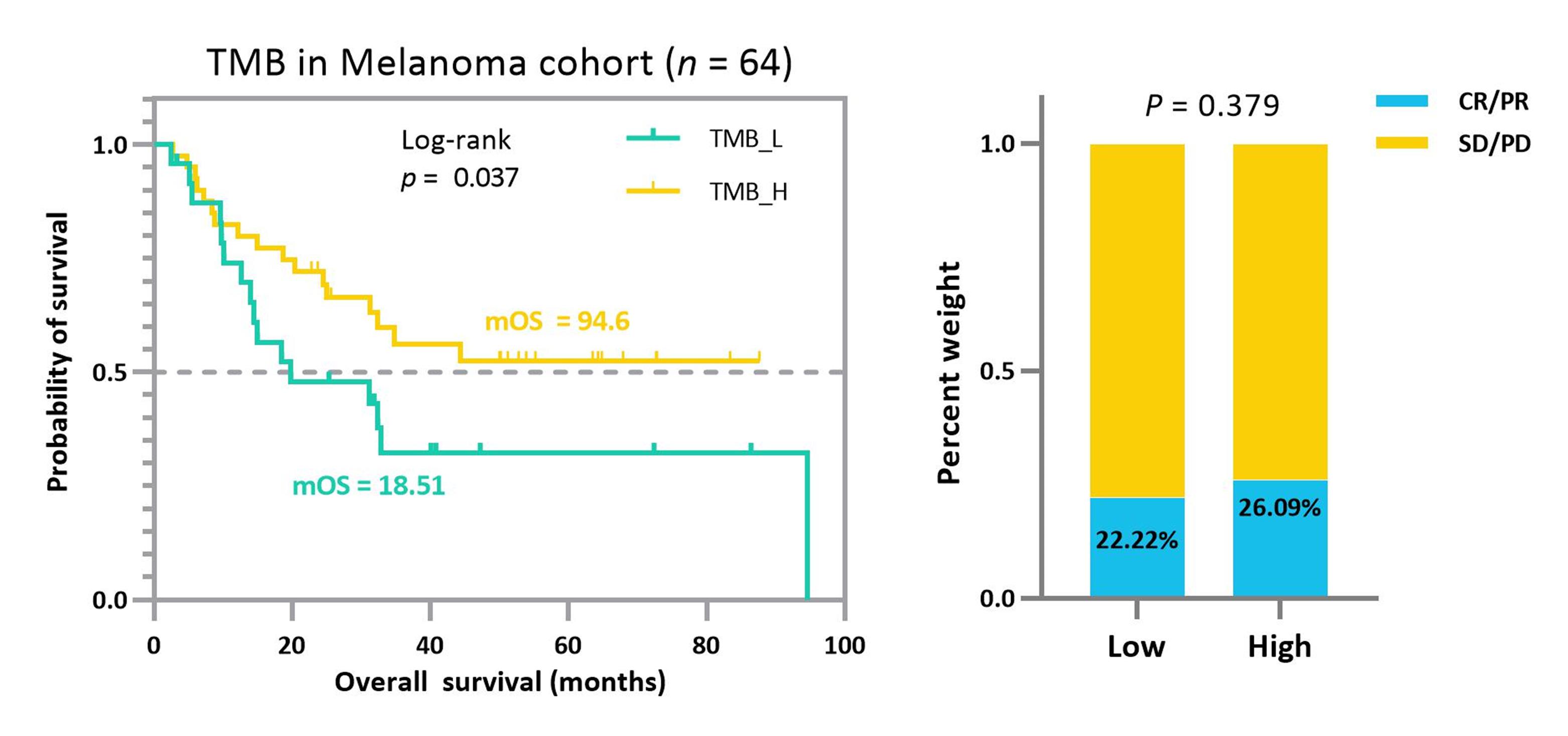

Supplement: Supplementary file 9 [file Image10.JPEG]

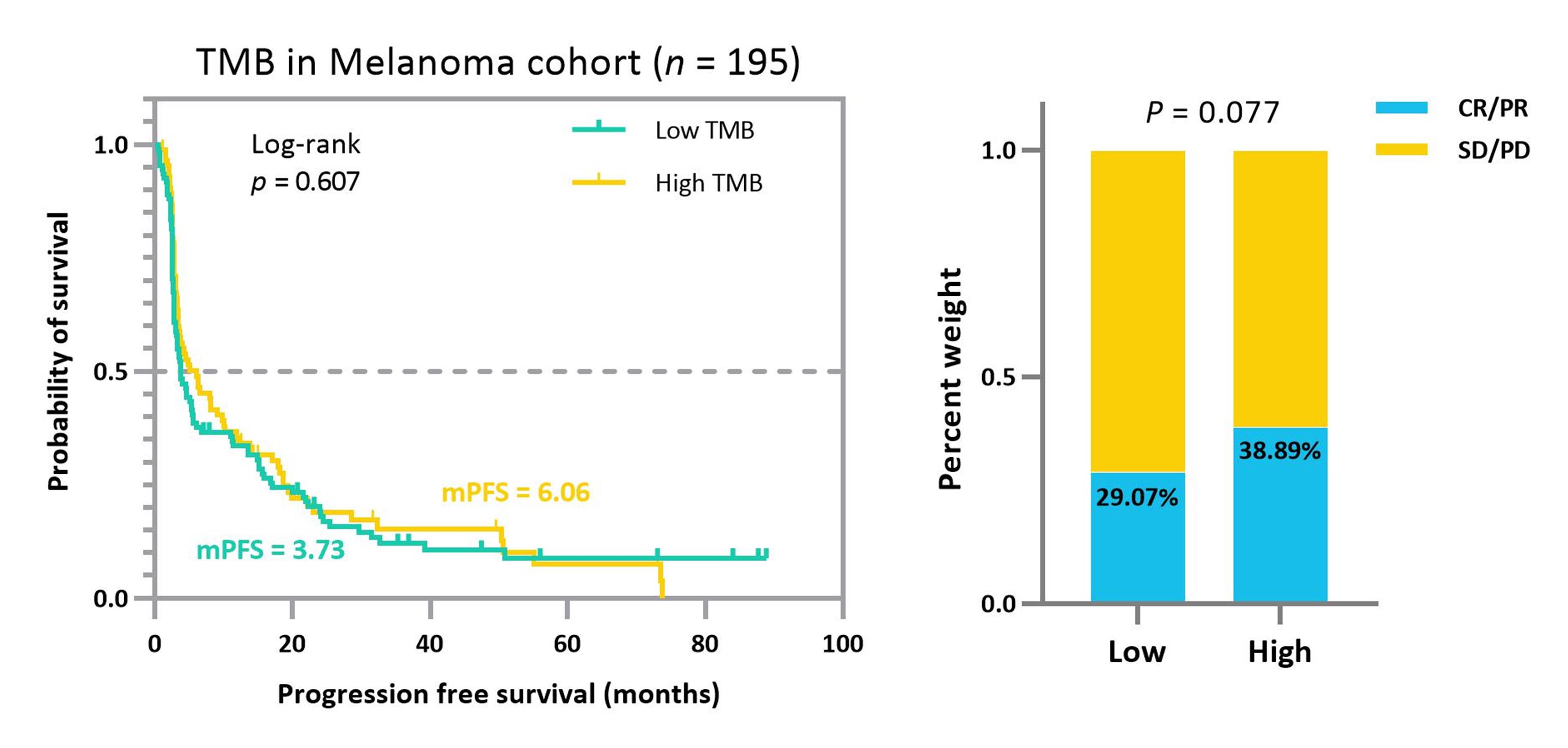

Supplement: Supplementary file 10 [file Image12.JPEG]

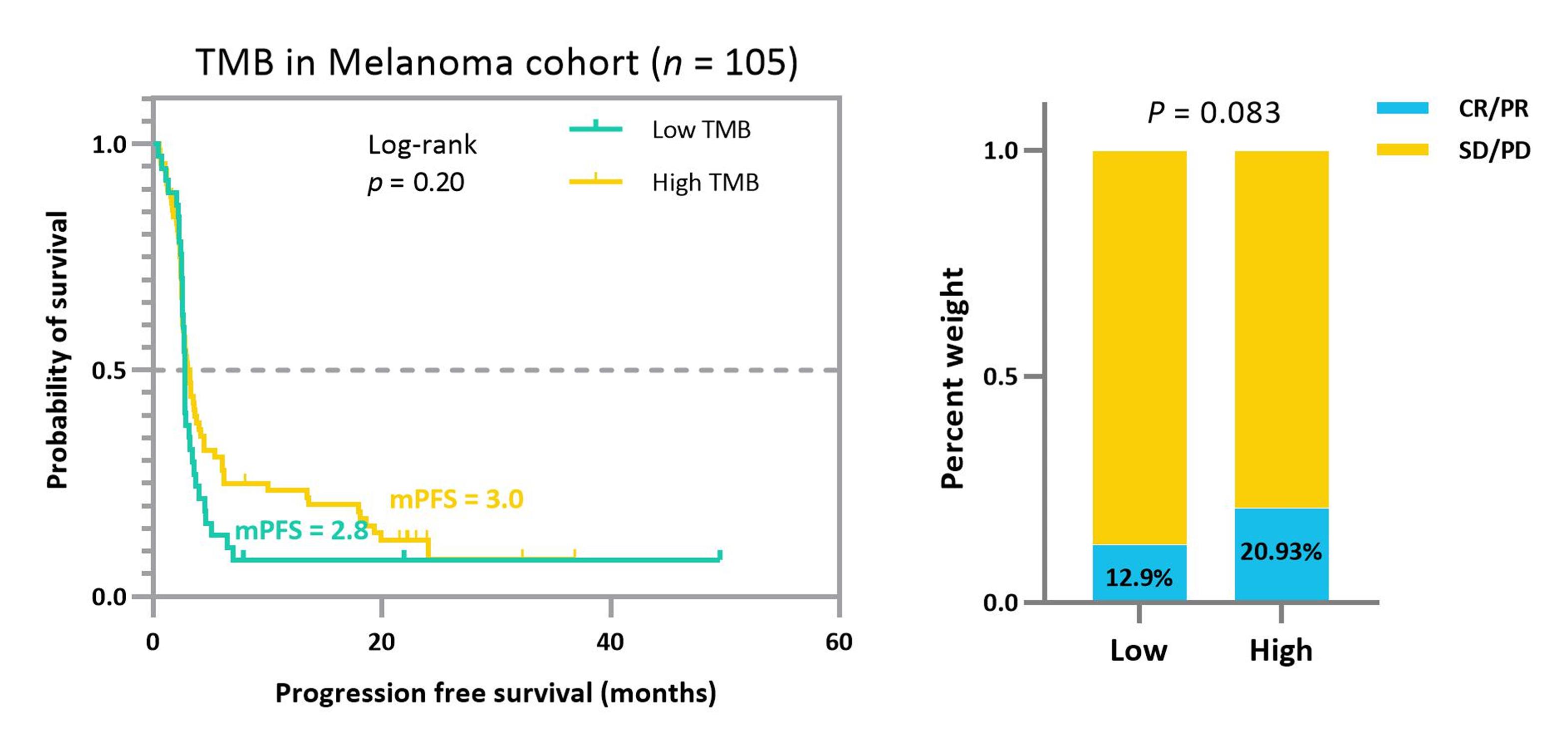

Supplement: Supplementary file 11 [file Image11.JPEG]

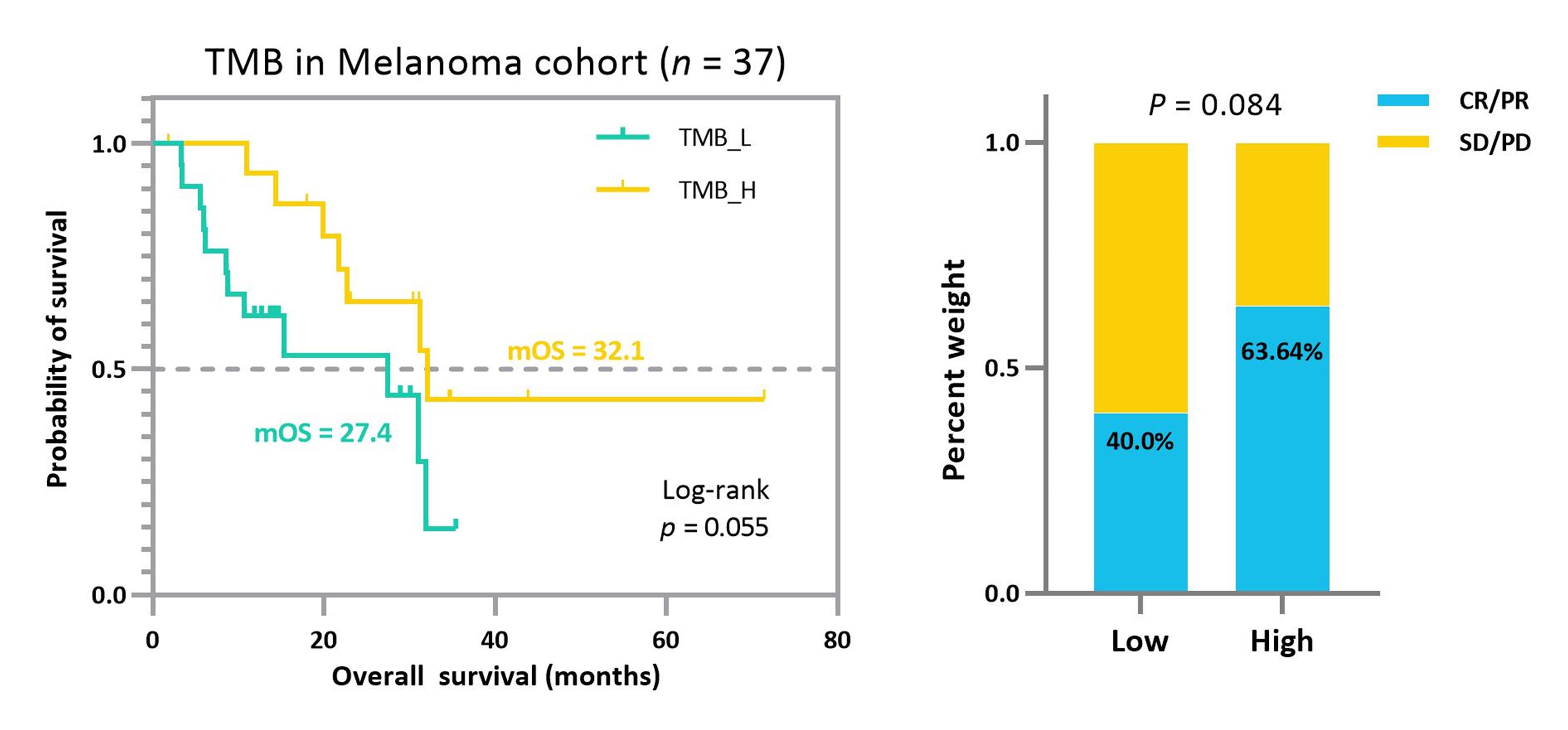

Supplement: Supplementary file 14 [file Image8.JPEG]

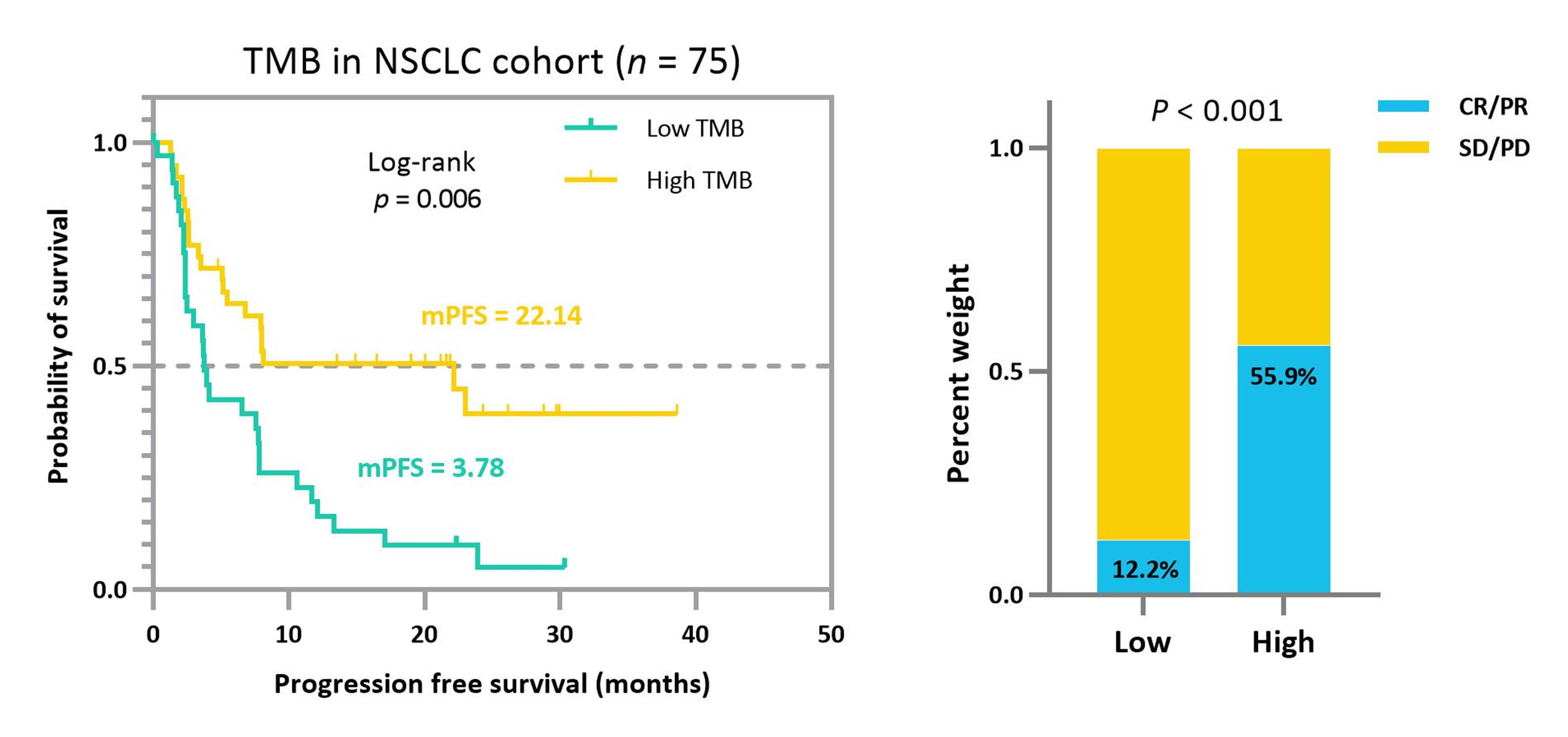

Supplement: Supplementary file 15 [file Image6.JPEG]
