# Supplementary material for: A Joint Model Considering Measurement Errors for Optimally Identifying Tumor Mutation Burden Threshold
Source: Front Genet. 2022 Aug 4;13:915839. doi: 10.3389/fgene.2022.915839 (PMC9386083; doi:10.3389/fgene.2022.915839)
Supplement: Supplementary file 12 [file DataSheet1.PDF]

Supplementary Material

1 Library preparation and NGS

Genomic DNAs from Formalin-fixed, paraffin-embedded (FFPE) or biopsy tumor samples and blood samples were extracted by QIAamp DNA FFPE Tissue Kit and DNeasy Blood and Tissue Kit (Qiagen, USA), respectively, and quantified by Qubit 3.0 using the dsDNA HS Assay Kit (ThermoFisher Scientific, USA) for whole-exome sequencing (WES). Next, a library was prepared with KAPA Hyper Prep Kit (KAPA Biosystems), and the target enrichment was performed using the xGen Exome Research Panel and Hybridization and Wash Reagents Kit (Integrated DNA Technology), followed by sequencing with Illumina HiSeq4000. For the targeted panel, customized xGen lockdown probes (Integrated DNA Technologies) targeting 422 cancer-relevant genes were used for hybridization enrichment. According to the manufacturers' protocols, the capture reaction was performed with Dynabeads M-270 (Life Technologies) and xGen Lockdown hybridization and wash Kit (Integrated DNA Technologies), and followed by sequencing on HiSeq4000. The average coverage depth was 140X and 1341X for tumors (64X and 143X in normal blood controls) using WES and Panel, respectively.

Then, FASTQ files were processed using Trimmomatic for quality control, with leading/trailing low quality (below 20) or N bases removed. The high-quality paired-end reads were aligned to the human hg19 reference genome using the Burrows-Wheeler Aligner (BWA). The resulting alignment files were cleaned by Picard and standardized GATK3, including deduplication, BQSR and Indel realignment. Cross-sample contamination was estimated using ContEst (Broad Institute, contamination rate <0.02). Somatic single nucleotide variant (SNV) and insertion/deletions (indels) calling was performed by Mutect and Scalpel, respectively. Variants were further evaluated at variant allele frequency (VAF) of ≥2 and support reads of ≥4, and annotated by vcf2maf. Tumor mutation burden for WES was defined as the total number of missense mutations. Panel TMB was calculated by adding all base substitutions and indels in the coding region of targeted genes. Synonymous variants were counted to reduce sampling noise, and known driver mutations were excluded due to over-representation, as described in the previous study (Chalmers et al., 2017).

2 The proofs of corrected-score function

Lemma 1. According to the arguments of Nakamura, 1990, we have the following equivalence properties. ℓ_c^* which satisfies $\mathbf{E}\{\ell_c^*(\theta,\mathbf{Z}_i,TMB_i^*)|TMB_i\}=\ell(\theta,\mathbf{Z}_i,TMB_i)$ is called *corrected-likelihood*. Under typical regularity conditions, $\mathbf{E}$ and $\partial\theta$ is interchangeable, then the derived score function $\Psi_c^*(\theta)=\frac{\partial\tilde{\ell}_c^*(\theta)}{\partial\theta^T}$ is the corrected-score. Moreover, the derived Hessian matrix is called a corrected observed Hessian, $H_c^*(\theta)=-\frac{\partial\Psi_c^*(\theta)}{\partial\theta^T}$, and $\mathbf{E}\{H_c^*(\theta,\mathbf{Z}_i,TMB_i^*)|TMB_i\}=H(\theta,\mathbf{Z}_i,TMB_i)$.

Lemma 2. Under the additive error model, the corrected likelihood for survival part is

$$\ell_{T\_c,i}^*(\theta)=\Delta_i[\log\{h_0(T_i)\}+(\beta_z^T\mathbf{Z}_i+\beta_m TMB_i^*+b_i)]-\mathcal{H}_0(T_i)\exp(\beta_z^T\mathbf{Z}_i+\beta_m TMB_i^*+b_i)m(\beta_m)^{-1}$$

where $m(t)=E[\exp(t\cdot e_i)]$ is the moment generating function of error term e_i.

Proof.

$$\begin{aligned}
&\mathbf{E}\{\ell_{T_c}^*(\theta,\mathbf{Z}_i,TMB_i^*)|TMB_i\}\\
&=\mathbf{E}\{\Delta_i[\log\{h_0(T_i)\}+(\beta_z^T\mathbf{Z}_i+\beta_m TMB_i^*+b_i)]-\mathcal{H}_0(T_i)\exp(\beta_z^T\mathbf{Z}_i+\beta_m TMB_i^*+b_i)m(\beta_m)^{-1}|TMB_i\}\\
&=\Delta_i\left[\log\{h_0(T_i)\}+\left(\beta_z^T\mathbf{Z}_i+\beta_m(TMB_i+\mathbf{E}\{\beta_m e_i|\mathbf{Z}_i,TMB_i\})+b_i\right)\right]\\
&\quad-\mathcal{H}_0(T_i)\exp(\beta_z^T\mathbf{Z}_i+\beta_m TMB_i+b_i)m(\beta_m)^{-1}\mathbf{E}\{\exp(\beta_m e_i)\,|TMB_i\}
\end{aligned}$$

Given the independency assumption of error term e_i

$$\mathbf{E}\{\beta_m e_i|TMB_i\}=\mathbf{E}\{\beta_m e_i\}=0$$

$$\mathbf{E}\{\exp(\beta_m e_i)\,|TMB_i\}=\mathbf{E}\{\exp(\beta_m e_i)\}=m(\beta_m)$$

$$\mathbf{E}\{\ell_{T\_c}^*(\theta,\mathbf{Z}_i,TMB_i^*)|TMB_i\}=\ell_T(\theta,\mathbf{Z}_i,TMB_i).$$

Unfortunately, the logistic distribution function $F(v)=(1+e^{-v})^{-1}$ cannot intuit the exact corrected-score function mathematically because it does not satisfy the smoothness conditions. Therefore, we consult the corrected score via complex variable simulation extrapolation proposed by Novick, 2011.

Lemma 3. Based on the complex variable theory and Monte Carlo approximation, we derive the following scheme:

i. For $j=1,...,J$, considering the complex variate $\widetilde{TMB}_{j,i}^*=TMB_i^*+\sqrt{-1}\xi_{j,i}$, where $\xi_{j,i}$ is a normal random vector with zero mean and variance σ_e.
ii. Define the Monte Carlo corrected score

$$\Psi^*_{MC,J}(R_i,T_i,\Delta_i,\mathbf{Z}_i,TMB_i^*;\boldsymbol{\Theta}) = J^{-1}\sum_{j=1}^{J}\text{Re}\left\{\Psi\left(R_i,T_i,\Delta_i,\mathbf{Z}_i,\widetilde{TMB}^*_{j,i};\boldsymbol{\Theta}\right)\right\}$$

iii. As $J \to \infty$, $\Psi^*_{MC,J} \to \Psi^*_c$, the Monte Carlo corrected score converges to the exact conditional expectation desired.

$$\mathbf{E}_\theta\{\Psi^*_{MC,J}(R_i,T_i,\Delta_i,\mathbf{Z}_i,TMB_i^*;\boldsymbol{\Theta})\,|TMB_i\} = \Psi(R_i,T_i,\Delta_i,\mathbf{Z}_i,TMB_i;\boldsymbol{\Theta}).$$

The number of generated complex variates per subject, *J*, needs to be large enough to make the limit approximately correct. However, for measurement error variances of the magnitudes commonly encountered in applications, rather small values of *J* suffice.

Proof of $k_c'(b_i)$

$$k_c''(b_i) = J^{-1}\sum_{j=1}^{J}\frac{\partial\text{Re}\left\{F\left(\alpha_z^T\mathbf{Z}_i+\alpha_m,\widetilde{TMB}^*_{j,i}+b_i\right)\right\}}{\partial b_i} - T_i^{\lambda} m(\beta_m)^{-1}\exp(\beta_z^T\mathbf{Z}_i+\beta_m TMB_i+b_i) - \sigma_b^{-2}$$

Given Lemma 1 and Lemma 2, corrected likelihood function is equivalent to corrected score function, then:

$$\mathbf{E}\{k_c''(b_i)\,|TMB_i\} = k''(b_i)$$
$$\mathbf{E}\{\Psi^*_{c,i}(R_i,T_i,\Delta_i,\mathbf{Z}_i,TMB_i^*;\boldsymbol{\Theta})\,|TMB_i\} = \Psi_i(R_i,T_i,\Delta_i,\mathbf{Z}_i,TMB_i;\boldsymbol{\Theta}).$$

Proof of Ψ^*_c

The Ψ^*_c is the corrected-score function follows easily from Lemma2 and Lemma3.
